# What Is New for an Old Molecule? Systematic Review and Recommendations on the Use of Resveratrol

**DOI:** 10.1371/journal.pone.0019881

**Published:** 2011-06-16

**Authors:** Ole Vang, Nihal Ahmad, Clifton A. Baile, Joseph A. Baur, Karen Brown, Anna Csiszar, Dipak K. Das, Dominique Delmas, Carmem Gottfried, Hung-Yun Lin, Qing-Yong Ma, Partha Mukhopadhyay, Namasivayam Nalini, John M. Pezzuto, Tristan Richard, Yogeshwer Shukla, Young-Joon Surh, Thomas Szekeres, Tomasz Szkudelski, Thomas Walle, Joseph M. Wu

**Affiliations:** 1 Department of Science, System and Models, Roskilde University, Roskilde, Denmark; 2 Department of Dermatology, University of Wisconsin, Madison, Wisconsin, United States of America; 3 Department of Animal and Dairy Science, University of Georgia, Athens, Georgia, United States of America; 4 Department of Physiology and Institute for Diabetes, Obesity, and Metabolism, University of Pennsylvania School of Medicine, Philadelphia, Pennsylvania, United States of America; 5 Department of Cancer Studies and Molecular Medicine, The Biocentre, University of Leicester, Leicester, Great Britain, United Kingdom; 6 Reynolds Oklahoma Center on Aging, University of Oklahoma Health Sciences Center, Oklahoma City, Oklahoma, United States of America; 7 Cardiovascular Research Center, University of Connecticut School of Medicine, Farmington, Connecticut, United States of America; 8 INSERM U866, University of Burgundy, Dijon, France; 9 Department of Biochemistry, Postgraduate Programme of Biochemistry, Institute of Basic Health Sciences, Federal University of Rio Grande do Sul, Porto Alegre, Brazil; 10 Signal Transduction Laboratory, Ordway Research Institute, Albany, New York, United States of America; 11 Department of Hepatobiliary Surgery, First Affiliated Hospital of Xi'an Jiaotong University, Xi'an, Shaanxi Province, China; 12 Laboratory of Physiological Studies, Section on Oxidative Stress Tissue Injury, National Institutes of Health, Rockville, Maryland, United States of America; 13 Department of Biochemistry and Biotechnology, Annamalai University, Annamalainagar, Tamil Nadu, India; 14 College of Pharmacy, University of Hawaii at Hilo, Hilo, Hawaii, United States of America; 15 UFR Pharmacie, University of Bordeaux, Villenave d'Ornon, France; 16 Proteomics Laboratory, Indian Institute of Toxicology Research, Lucknow, Uttar Pradesh, India; 17 College of Pharmacy, Seoul National University, Seoul, South Korea; 18 Clinical Institute of Medical and Chemical Laboratory Diagnostics, General Hospital of Vienna, Medical University of Vienna, Vienna, Austria; 19 Department of Animal Physiology and Biochemistry, August Cieszkowski University of Agriculture, Poznan, Poland; 20 Department of Cell and Molecular Pharmacology and Experimental Therapeutics, Medical University of South Carolina, Charleston, South Carolina, United States of America; 21 Department of Biochemistry and Molecular Biology, New York Medical College, Valhalla, New York, United States of America; University of Valencia, Spain

## Abstract

**Background:**

Resveratrol is a natural compound suggested to have beneficial health effects. However, people are consuming resveratrol for this reason without having the adequate scientific evidence for its effects in humans. Therefore, scientific valid recommendations concerning the human intake of resveratrol based on available published scientific data are necessary. Such recommendations were formulated after the Resveratrol 2010 conference, held in September 2010 in Helsingør, Denmark.

**Methodology:**

Literature search in databases as PubMed and ISI Web of Science in combination with manual search was used to answer the following five questions: ^1^Can resveratrol be recommended in the prevention or treatment of human diseases?; ^2^Are there observed “side effects” caused by the intake of resveratrol in humans?; ^3^What is the relevant dose of resveratrol?; ^4^What valid data are available regarding an effect in various species of experimental animals?; ^5^Which relevant (overall) mechanisms of action of resveratrol have been documented?

**Conclusions/Significance:**

The overall conclusion is that the published evidence is not sufficiently strong to justify a recommendation for the administration of resveratrol to humans, beyond the dose which can be obtained from dietary sources. On the other hand, animal data are promising in prevention of various cancer types, coronary heart diseases and diabetes which strongly indicate the need for human clinical trials. Finally, we suggest directions for future research in resveratrol regarding its mechanism of action and its safety and toxicology in human subjects.

## Introduction

Resveratrol (Resv) is a simple molecule that has taken the spotlight since the first scientific paper described a possible preventive effect on cancer in mice [1]. Resveratrol occurs naturally in low amounts in various edible plants, but the fact that Resv is found in red wine increases its relevance as being easily accessible to the general population. The applications of Resv therefore receive strong attention from the general population, the scientific community and companies invested in food additives, cosmetics and “natural medicine.”

A number of long-term clinical studies in humans have recently been initiated or is under planning and ideally in 2–5 years, we will know much more about the biological effects of Resv in humans. But before these data are available, the prediction of biological effects of Resv in humans have to rely primarily on data obtained in experimental animals and from *in vitro* screening in combination with elucidation of its mechanism of action. All reliable Resv data should be included to convert knowledge generated in animals into a clinically safe Resv treatment approach in humans. Therefore, a critical evaluation of the present scientific state-of-the-art knowledge is needed.

The aim of the recent conference, Resveratrol2010, *1^st^ International Conference on Resveratrol and Health* (www.resveratrol2010.com), was to present the state-of-art of knowledge in the Resv field. After the conference invited speakers and the scientific committee formed a working group which formulated the recommendations described herein.

The task of the working group discussion was to formulate a number of scientifically based recommendations for ^1^the human use of resveratrol and ^2^research on resveratrol for the coming years based on scientific literature and data made available during the previous 2½ days of the conference. As Resv has been suggested to promote health in relation to various diseases or sufferings, the participating scientists covered a broad range of research on the biological effects of Resv, which included the following subjects, ^1^resveratrol and cancer; ^2^resveratrol and heart disease; ^3^neuroprotective activity of resveratrol; ^4^effect of resveratrol on longevity; ^5^effect of resveratrol on inflammation; ^6^effect of resveratrol on obesity and diabetes; ^7^metabolism and stability of resveratrol and ^8^production and commercial use of resveratrol.

The final recommendations to the use of resveratrol are given in Tables 1, 2, 3, 4, 5, 6, whereas Table 7 describes recommendations for research goals for the coming years in this field. The recommendations to the use of resveratrol do not differentiate between Resv used as a drug (for disease treatment), as part of food or as food supplement (maintenance of good health). On the other hand, Resv consumption as a food item or food supplement is considered as the primary area.

**Table 1:** Recommendations for the use of resveratrol – part 1.

Can resveratrol be recommended in the prevention or treatment of human diseases?
• There are not yet unequivocal scientific data for the effect of resveratrol as a disease preventative substance in humans nor for human life extension.
• There is not yet sufficient evidence for a therapeutic effect of resveratrol in humans, either alone or in combination with other natural compounds or formulations.

**Table 2:** Recommendations for the use of resveratrol – part 2.

Are there observed “side effects” caused by the intake of resveratrol in humans?
• There are no valid data on the toxicity of chronic intake of resveratrol in humans.
• A short term human study (29 days) indicated frequent gastrointestinal discomfort/diarrhea only at high doses (2.5 g or 5 g per day). Only minor and inconsistent side effects have been observed in other short-term or acute studies.

**Table 3:** Recommendations for the use of resveratrol – part 3.

What is the relevant dose of resveratrol?
• A relevant or optimal dose for resveratrol has yet to be established by human studies and will almost certainly vary depending on the effect being studied.
• Doses in the range of hundreds of mg to several g per day have been proposed based on animal studies, but more human studies are needed to confirm these estimates.
• Chronic human intake above the concentrations contained in natural food should be considered experimental until long-term human studies have been performed.

**Table 4:** Recommendations for the use of resveratrol – part 4.

What valid data are available regarding an effect in various species of experimental animals?
• There is sufficient evidence for a chemopreventive effect of resveratrol on the development of cancer in skin of mice. There are promising results on the prevention of colon cancer in animals. The effects of resveratrol on other cancer types than skin cancer need to be investigated more in detail prior to recommending clinical trials.
• There is sufficient evidence to suggest resveratrol reduces the incidence of hypertension, heart failure, ischemia heart disease in experimental animal models.
• There is sufficient evidence to suggest resveratrol improves insulin sensitivity, reduces blood glucose levels, and reduces high fat diet-induced obesity in rodents.
• Resveratrol showed neuroprotective effects in experimental animal models of injury or degeneration.
• Resveratrol is well tolerated in rats and no toxicological effects are observed up to 700–1000 mg/kg bw/day.

**Table 5:** Recommendations for the use of resveratrol – part 5.

Which relevant (overall) mechanisms of action of resveratrol have been documented?
• Modulation of cell proliferation and apoptosis
• Modulation of angiogenesis
• Inhibition of metastasis
• Modulation of redox status
• Suppression of adipogenesis and stimulation of adipocyte lipolysis
• Stimulation of osteogenesis
• Modulation of mitochondria activity
• Suppression of inflammation
• Modulation of DNA damage
• Modulation of xenobiotic metabolism
• Modulation of glutamate metabolism
• Estrogenic activity/anti-estrogenic activity

**Table 6:** Overall conclusions for the use of resveratrol.

1	Published evidence today is not sufficiently strong to justify recommendation for the chronic administration of resveratrol to human beings, beyond the dose which can be obtained from dietary sources.
2	Animal data are promising and indicate the need for further human clinical trials.

**Table 7:** Recommendations for research on resveratrol for the coming years.

1	Clinical studies should be initiated, especially with focus on the effect of resveratrol on the development of cancers in colon and skin
2	Clinical studies should be initiated to test the potential cardio-vascular benefit of resveratrol
3	Elucidating the biological effects of resveratrol metabolites
4	Biodistribution and degradation of resveratrol *in vivo*, including the role of bacterial enzymes
5	Preparation of a resveratrol reference (international standard) product for analytical purposes
6	Standardized formulations for clinical studies
7	Combinatory effects of resveratrol with other compounds. This include development of relevant models
8	Interaction of resveratrol with drug metabolism (especially cytochrome P450 metabolism)
9	Identification/development of relevant biomarkers, relevant for the disease-prevention rather than disease treatment, depending on the relevant disease
10	Effect of resveratrol on inflammation as general condition relevant for several lifestyle diseases
11	Long term preclinical studies in nonhuman primates may be appropriate to determine the effect of resveratrol on diet-induced metabolic disorders, such as development of insulin resistance

## Methods

To generate a scientifically valid foundation for the formulation of these recommendations, a systematic search was performed in MEDLINE (http://www.ncbi.nlm.nih.gov/pubmed/) and ISI Web of Science (http://apps.isiknowledge.com/) to indentify studies in humans or experimental animals on Resv in relation to cancer, coronary heart disease, diabetes/metabolic syndrome, neurodegenerative diseases and inflammation up to September 2010. The search terms “resveratrol” was used in combination with “cancer”, “carcinogenesis”, “chemoprevention”, “hypertension”, “heart failure”, “myocardial infarct”, “cardiac arrest”, “ischemia heart disease”, “stroke”, “serum lipids”, “metabolic syndrome”, “obesity”, “diabetes”, “insulin sensitivity”, “plasma glucose”, “visceral fat”, “neuroprotection”, “neuroprotective”, “inflammation” or “inflammatory”, were used to search for words in titles, abstracts, or Medical Subject Headings. The search was limited to English language but include both human data and experimental animals. Beside the database searches a manual search was performed using reference lists of original articles and previous reviews. For all studies, only the original publication was included in the present evaluation. Only studies investigating Resv and Resv metabolites are evaluated whereas various derivatives of resveratrol were not included.

The identified publications were used to answer the following five questions: ^1^Can resveratrol be recommended in the prevention or treatment of human diseases?; ^2^Are there observed side effects caused by intake of resveratrol in humans?; ^3^What is the relevant dose of resveratrol?; ^4^What valid data are available regarding an effect in various species of experimental animals?; ^5^Which relevant (overall) mechanisms of action of resveratrol have been documented?

## Results

**Figure 1:**
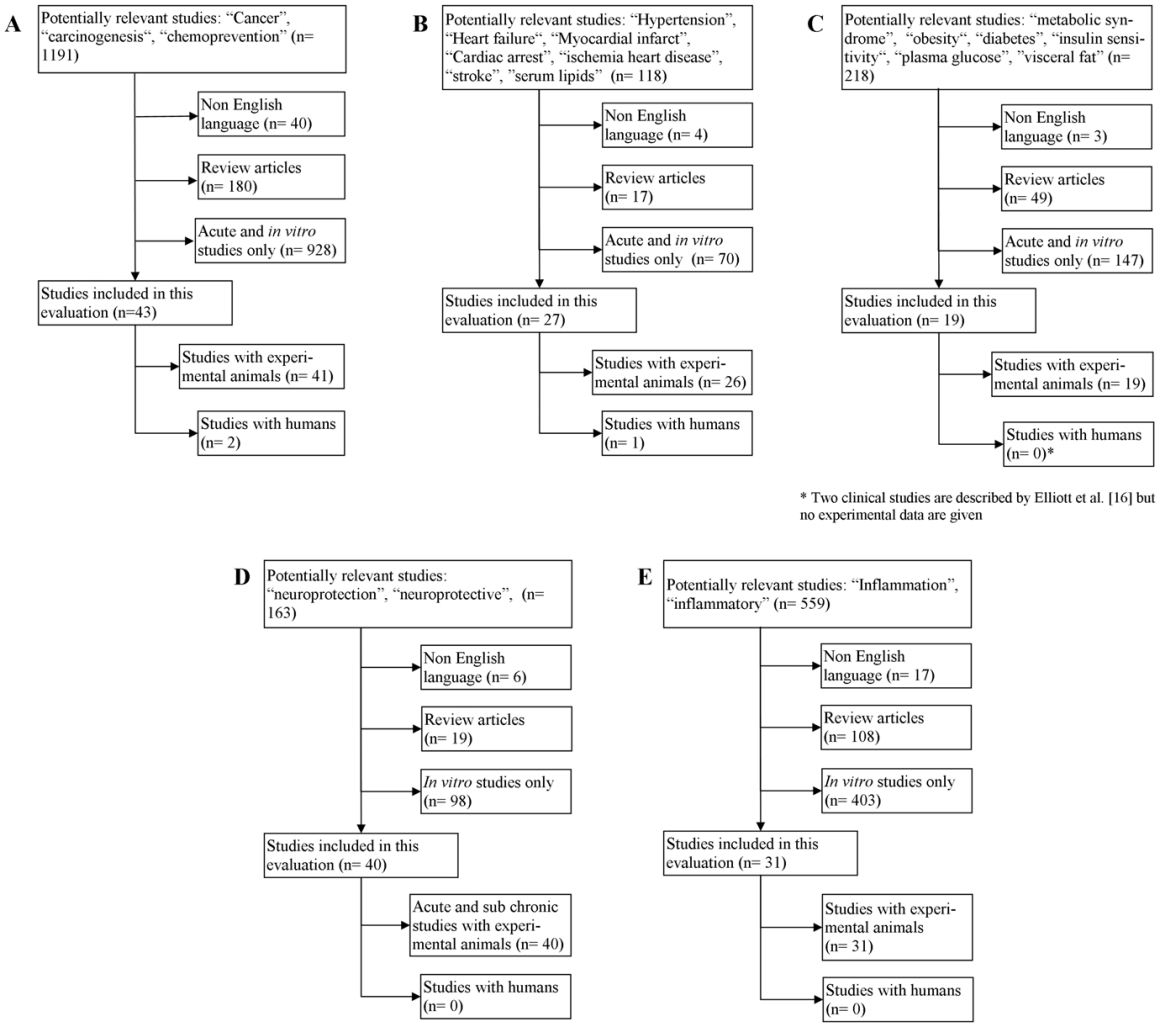
Flow chart of systematic literature search. The literature search was performed to identify all relevant articles focusing on resveratrol and chemoprevention (A), effect of resveratrol on cardio vascular disease (B), effect of resveratrol obesity and diabetes (C), neuroprotective effect of resveratrol (D) and the anti-inflammatory effect of resveratrol (E). In all five groups, articles with non-english language, review articles and articles showing data from *in vitro* experiments only are excluded. For chemopreventive effect (A), cardio vascular disease (B), obesity and diabetes (C), also non-chronic animal studies were excluded.

The term “resveratrol” is found in title, abstract or MESH word in 5425 or 3650 publications following searching on ISI Web of Science or PubMed, respectively. The substantially higher number of hits found in ISI Web of Science was due to abstracts or papers with focus on identification of sources of Resv. All the publications relevant for this systematic review were present in both data bases and the refinement of the search using PubMed is indicated in Figure 1. To identify the cancer preventive potential of Resv, in total 1191 papers were identified (Figure 1A), but by excluding non English papers, reviews and papers analyzing acute or *in vitro* effects only, 41 articles were found to test Resv in animal model systems, whereas two studies focused on the effect of Resv in human subjects. The effect of Resv on coronary heart diseases was investigated in 118 papers (Figure 1B); whereas only 26 papers show data from non acute animal experiments and one paper was identified investigating effect in humans. Focusing on the effect of Resv on obesity and related diseases such as diabetes, 218 articles were identified (Figure 1C), but only 19 animal studies and no human studies focused on non-acute effects of Resv. Neuroprotection by Resv was in focus of 163 papers (Figure 1D), but 40 papers concerned animal studies, including both acute exposure and sub- and chronic studies. Lastly, 559 papers focused on resveratrol and inflammation (Figure 1E), and of these 31 papers included sub chronic and chronic studies, whereas no human studies focusing on inflammation have been published.

## Discussion

### Can resveratrol be recommended in the prevention or treatment of human diseases?

A major challenge for scientists investigating Resv is to prove that it has the health promoting effects, which have been suggested based on the *in vitro* and animal studies available. Clinical trials with Resv in human subjects focusing on the health promoting effect of Resv are lacking. Therefore, these studies have the highest priority in recommendations from the scientific working group (Table 7). Two clinical trials have been recently published analyzing the effect on biomarkers of intermediary metabolism: 2.5 g Resv/day for 29 days was found to significantly reduce the plasma level of insulin-like growth factor-1 and insulin-like growth factor binding protein-3 indicating a possible cancer preventive effect [2], whereas daily doses of 0.5 and 1.0 g Resv/day for 29 days caused a reduction of cell proliferation in colon cancer tissue [3].

Most of the available clinical studies with Resv in humans focus on bioavailability, pharmacokinetics and metabolism of Resv [4]–[11]. These studies showed that Resv was rapidly absorbed after oral intake; a maximal plasma concentration of Resv obtained after 30 to 60 minutes. Further, the level of Resv in the blood stream was low, likely caused by rapid metabolism to glucuronide and sulphate conjugates. In addition to the studies described above, levels of Resv after ingestion of Resv-containing items such as wine or grape juice have been investigated [12]–[15]. However, the specific effect of Resv is difficult to estimate when given as part of food matrices.

Two clinical studies were described by Elliott *et al.*, where the effect of Resv on type 2 diabetic patients was tested at 2.5 or 5.0 g/day for 28 days [16]. The levels of fasting and postprandial plasma glucose and postprandial serum insulin were statistically significantly decreased with 5.0 g/day, but the experimental details were not given.

The tissue distribution of Resv and its metabolites was estimated in a study where twenty colon cancer patients received 0.5 g or 1.0 g Resv daily for eight days before surgical resection [3]. The level of Resv was found in colon tissue at a level sufficient to elicit anticarcinogenic effects observed in *in vitro* tests. A recent experiment showed that single doses of 250 and 500 mg Resv significantly increased cerebral blood flow but the cognitive performance was unaltered [17].

Several clinical trials of oral Resv as a pure compound or using Resv-rich products (grapes and grape juice) are under way. In total, 24 clinical studies are listed at the homepage http://clinicaltrials.gov/ [18] having Resv as the major experimental subject or as part of a clinical trial. Therefore, it is our recommendation that clinical trials should focus on biomarkers of cancers, diabetes and metabolic syndromes as well as neurological diseases (Table 7).

Based on the limited number of human studies, the working group has concluded that there is not at the moment enough available and scientifically valid data on the effect of Resv to conclusively state whether it could be a disease preventive substance in humans or could be used for human life extension (Table 1). It is therefore vital to perform such studies.

### Are there observed side effects caused by the intake of resveratrol in humans?

Based on animal studies, Resv is generally well tolerated, and only very few short-term or acute exposure experiments in humans have been performed. When eight healthy subjects were exposed to 2000 mg Resv twice/day for eight days, six of eight subjects had mild episodic diarrhea/loose stool, typically in the beginning of the eight days treatment period, and one of the subjects developed a temporary rash and headache [9]. In a double-blinded, randomized, placebo-controlled study, up to 975 mg/day were given to healthy volunteers, where two adult subjects (male and female) in each group were subjected to 25, 50, 100 or 150 mg, six times/day, for two days in total. Adverse effects were mild in severity and similar between all groups. Repeated administration of Resv was well-tolerated but produced relatively low plasma concentrations of Resv, despite the high doses and short dosing interval used [10]. Exposure of up to 270 mg Resv to 19 volunteers for one week did not cause any discomfort [19].

According to Elliott *et al*. healthy volunteers tolerated Resv well in a seven-day exposure study, but experimental details were not provided thus making evaluation of results challenging [16]. The same article describes very briefly a study which included daily exposure to 2.5 g or 5 g Resv for 28 days. The authors reported that “Adverse events were generally mild in nature and reversible” but no experimental details are shown, which made a closer evaluation impossible [16]. The 20 colon cancer patients receiving 0.5 g or 1.0 g Resv daily for eight days before surgical resection [3] tolerated it well.

It is difficult to estimate the normal human consumption of Resv as the intake of red wine (verified main source of Resv) differs greatly in the population and the content of Resv varies (mean 1.9±1.7 mg/L) [20], but the dose may be up to 4 mg/person/day.

Only a single experiment has tested Resv in a classical chronic exposure experiments, i.e. at least 24 months in rats or 18 months in mice [21] and Resv does not in general cause any toxic effects in animal studies published except at doses above 1 g/kg bw/day. Besides acute exposure to Resv, several sub chronic experiments have indicated low toxicity. In male Sprague-Dawley rats, 20 mg Resv/kg bw/day given for 28 days did not indicate systemic toxicity [22]. Oral doses of 300, 1000 or 3000 mg Resv/kg bw/day for 28 days only showed toxic effects in the 3000 mg treatment group (CD rats, both sexes) [23], where nephropathy and renal toxicity were observed as well as changed clinical markers of liver metabolism. Exposure of Wistar rats to Resv (50, 150 or 500 mg/kg bw/day) for four weeks or three months to 120, 300 or 700 mg/kg bw/day did not show significant toxicological effects [24]. An additional study of 28 days in rats is mentioned by Elliott *et al*., which showed “a no effect level” at 300 mg/kg bw/day [16] but no experimental details were given and the results could not be fully evaluated. CD rats (both sexes) exposed to 0, 300, 1000 or 3000 mg Resv/kg bw/day by gavages for 28 days showed only mild liver toxicity as well as nephrotoxicity when exposed to the highest dose [25]. In a study, where female Sprague-Dawley rats were exposed to Resv (1 g/kg diet, corresponding to about 100 mg/kg bw/day in an adult), for the entirety of their life starting at birth showed no toxicological signs such as reduced food intake, reduced body weight, or delayed sexual maturation [26].

In mice (C57BL/6 p53^−/−^) oral administration of Resv (1000, 2000, 3000, 4000 or 5000 mg/kg bw/day) showed an increased death rate caused by impaction of Resv in the gastrointestinal tract [27]. Long term administration of Resv in drinking water (14 mg/L water) to mice for six months cause a reduced organ weight but these differences were not present in a corresponding 12 month experiment [28].

Elliott *et al* [16] cites a study of Resv exposure to rabbits: Severe toxicity was observed at high doses with the kidney as the primary target. The ‘no effect level’ was estimated at 500 mg/kg bw/day in males and 250 mg/kg bw/day in females [16], but no further details were given in the paper. Likewise, Elliott *et al.* cites a study on the effect of Resv in dogs for 28 days, which showed no toxic effects at 300 mg Resv/kg bw/day, but no details were given [16].

Several experiments have shown that Resv does not have genotoxic activity, based on the Ames test [16], [24], but experimental details are too limited to evaluate the data fully. Supporting this, there was no increase in the frequency of micronucleated immature erythrocytes observed in rats exposed to up to 200 mg Resv/kg bw/day [24].

Reproductive toxicity was evaluated in rats and the maternal ‘no observed adverse effect level’ (NOAEL) was estimated at 300 mg/kg bw/day and the developmental NOAEL was estimated at 300 mg/kg bw/day, but no experimental details were given [16]. A study described by Williams *et al*, indicated that the NOAEL for maternal toxicity and embryo–fetal development was around 750 mg Resv/kg bw/day [24]. Based on these observations, the paper concluded that a daily dose of 450 mg was safe for a 60 kg person, using a 10 fold safety factor [24] which is further the basis of the self affirmed GRAS (Generally Recognized As Safe) status up to 450 mg Resv/day from several Resv producers.

Several examples of self reported side effects of Resv intake may be found on various homepages from the internet, but there is no comprehensive evaluation of these self reported side effects.

Based on the available data, which mostly originate from studies of very short duration, the working group formulated the following conclusions (Table 2):

- There are no valid data on the toxicity of chronic intake of resveratrol in human subjects.
- A short term human study (29 days) indicated frequent gastrointestinal discomfort/diarrhea only at high doses (2.5 g or 5 g per day). Only minor and inconsistent side effects have been observed in other short-term or acute studies.

### What is the relevant dose of resveratrol?

Resveratrol has been proposed to be active in the prevention of various life style diseases such as cancer, coronary heart diseases and type 2 diabetes. Different mechanisms are likely involved besides the modulation of inflammation as a unifying mechanism. Because of different mechanisms and targets one must assume that the optimal dose will depend on the particular disease.

As indicated above, only a few human studies have been performed, showing down-regulation of cancer biomarkers by 2.5 g Resv/day for 28 days [2] and reduced cell proliferation in colon cancer tissue was observed in humans at doses of 0.5 and 1.0 g Resv/day [3]. A single double-blind, randomized cross-over human study focused on the effect of Resv on flow-mediated dilatation of the brachial artery (as a biomarker of reduced endothelial function and cardiovascular health) in overweight/obese men and women: Resv, at sub chronic doses of 30, 90 and 270 mg/day for one week, showed a significant dose-dependent increase in flow-mediated dilation, significant even at the 30 mg dose [19].

Several human studies have investigated the effects of Resv containing food items. The exposure to Resv is likely in all cases less than 4 mg. Human acute or sub chronic (two weeks) intake of red wine, dealcoholized red wine or red grape juice did not show effect on Tumor necrosis factor (TNF) α, Interleukin (IL)-2 or IL-4 levels, indicating no anti-inflammatory response of the wine but the presence of Resv in the wine was not proven [29], [30]. Exposure of healthy volunteers to 36 µg Resv/day (combination of *trans*- and *cis*-Resv and *cis*-piceid) in the form of Chardonnay cava wine for 28 days caused a reduction of various inflammatory markers such as IL-6, high-sensitivity C-reactive protein (CRP), intercellular adhesion molecule-1 (ICAM-1) and monocyte chemoattractant protein-1 (MCP-1) [31].

Resveratrol is found to reduce the risk of colon cancer development in experimental animals (see below) using doses in the range of 0.2 to 8 mg/kg in rats and 2.4 to 60 mg/kg in mice. Applying the dose translations factors described by Reagan-Shaw *et al*. [32] the corresponding daily ‘human equivalent doses’ (HED) are around 2 mg–78 mg (based on rat data) or 12–290 mg (based on mouse data) for a 60 kg person.

Enhanced insulin sensitivity by Resv was observed in experimental animals when exposed to 2.5–400 mg/kg in mice and 1–100 mg/kg in rats (see below). The corresponding HED are 12–1945 mg (based on mouse data) or 10–973 mg/day for a 60 kg person.

The relevant effective dose of Resv needs to be established in humans in relation to the different diseases that it may counteract and the working group concludes (Table 3) that:

- A relevant or optimal dose for resveratrol has yet to be established by human studies and will almost certainly vary depending on the effect being studied.
- Doses in the range of hundreds of mg to several g per day have been proposed based on animal studies, but more human studies are needed to confirm these estimates.
- Chronic human intake above the concentrations contained in natural food should be considered experimental until long-term human studies have been performed.

### Which valid data is available regarding an effect in various species of experimental animals?

#### Cancer preventive activity

The initial paper by Jang *et al.* showed a cancer preventive effect of Resv on skin cancer in a mice model. Seven animal studies have been identified studying the effect of Resv on skin cancer (Table S1) all showing reduction of the incidence of chemically induced skin cancer [1],[33]–[36], whereas no reduction was observed in a mouse line spontaneous to developing polyps [37]. Only mice have been used in these studies with different Resv concentrations, dose regimens and exposure times (up to 28 week). A recent study supported this by showing reduction of skin hyperplasia by Resv alone and in synergism with other dietary components [38].

To test the effect of Resv on breast cancer, four studies used mice where Resv showed a reducing effect in three experiments [1], [39], [40] (in chemical induced cancers, in a HER-2/neu transgenic mice model and injected Ehrlich ascites carcinoma cells), whereas Resv failed to show chemoprevention in a model where 4T1 mammary carcinoma cells were injected into the mice [41]. In rats, three studies showed a chemopreventive effect of Resv [26], [42], [43], whereas increased breast cancer incidence is observed in one study [44]. The reason for these contradictory results is not obvious.

Resveratrol reduced chemically-induced liver cancer in rats [45] and in mice injected with carcinoma cells [46]–[48]. A single experiment showed no reduction of the incidence of esophagus carcinoma formation by Resv [49]. The effect of Resv on gastric cancers was evaluated with its effect on tumor growth after subcutaneous injection of cancer cells. Both studies in mice showed a reduced tumor volume as a consequence of Resv exposure [50], [51].

To study the effect of Resv on colon cancer development in animals, four studies focus on chemically-induced colon cancer in rats or mice [52]–[55] and three studies evaluated the effect of Resv on colon cancer in APC/Min mice strains [56]–[58]. Three of the tests showed significant reduction of the aberrant crypt formation or incidence of adenomas, whereas two of the mouse studies did not show a significant reduction in colon cancer incidence or tumor load.

Resveratrol showed a chemopreventive effect on development of prostate cancer using rat or mice strains prone to spontaneously developing prostate cancer [59]–[61] and a mouse model with injected prostate cancer cells [62] In other studies focusing on lung carcinogenesis, Resv showed a chemopreventive effect only in a single experiment out of a total of four experiments [63]–[66]. A single study focused on the effect of Resv on the development of neuroblastoma in mice, and found reduced tumor volume [67].

#### Cardioprotective effect of resveratrol

Risk reduction of cardiovascular events is one of the most well-known health promoting effects of Resv. It has been shown that Resv may modulate various aspects of cardio-vascular diseases, including atherosclerosis, hypertension, ischemia reperfusion injury and heart failure.

Resveratrol reduced hypertension in various models (Table S2), including spontaneously hypertensive rats [68], [69], salt induced hypertension [70], hypertension induced by monocrotaline [71], [72], or obesity-induced hypertension [73]–[76]. In nine out of eleven studies, Resv (at levels of 10 mg/kg bw/day or higher) was found to reduce the elevated blood pressure. The blood pressure reducing effect of Resv was observed after 3 weeks and maintained for 10 weeks. Low exposure to Resv (2.5 mg/kg bw/day for 10 weeks) was described in two experiments using spontaneous hypertensive rat where one experiment showed a preventive effect on hypertension whereas the other did not.

Several studies (five have been identified) showed a preventive effect of Resv on myocardial infarction induced by surgery. All studies showed a reduced infarct size effect and one experiment even at the low dose of 1 mg Resv/kg bw/day for four weeks [77]–[81]. Chronic treatment with Resv reduced infarct area after middle cerebral artery occlusion [82]. The diabetes-induced myocardial infarct size was significantly reduced by Resv (ranging from 1 to 5 mg/kg bw/day) in rats exposed to Resv for 5–15 days [83]–[85]. Further, Resv may precondition the heart in a nitric oxide (NO)-dependent manner, which would reduce heart damage from ischemia [85]. Resveratrol prevented the effects of ischemia at doses of 10 mg/kg, whereas higher doses were found to depress cardiac function and increase myocardial infarct size [86], [87]. A single experiment was performed in swine where the reduced inferolateral function induced by hypercholesterolemic diet was prevented by 100 mg Resv/kg bw/day [88].

#### Obesity and type 2 diabetes

Animal studies focusing on the effect of Resv on obesity and diabetes are shown in Table S3. Some studies have indicated that intake of Resv may reduce body weight increase caused by high fat intake in mouse [89] and grey mouse lemurs [90] both exposed to high levels of Resv (200 or 400 mg/kg bw/day). Low doses, up to 60 mg/kg bw/day did not show any effect on body weight [21], [73], [76], [91]. On the other hand, low doses did affect lipid accumulation observed as reduced size of white adipose tissue [91], reduced abdominal obesity [92], reduced abdominal fat in obese rats [73] and reduced grade of steatosis [93].

The effect of Resv on insulin sensitivity has been the subject in nine studies using rats or mice in which insulin sensitivity was reduced by high fat diets, using animal strains prone to developing insulin resistance or by chemically induced diabetes (treatment with streptozotocin). Nearly all experiments showed a reduced insulin level or increased insulin sensitivity using doses covering 2.5–400 mg/kg bw/day and exposure time covering 1–6 months [21], [73], [89], [92], [94], [95]. One study, using AMPK^α1−/−^ and wild-type C57BL/6J mice fed a high-fat diet and exposed to 400 mg Resv/kg bw/day for 12 weeks found an effect of Resv on insulin sensitivity in wild-type, but not AMPK^α1−/−^ mice [96]. In C57BL/6 male mice fed a high-calorie diet and a low dose of Resv (79.2 ng/day, infused intra-cerebro ventricularly) for five weeks, reduced the serum insulin levels significantly [97], indicating that the active dose of Resv for prevention of elevated insulin levels is low and may be mediated the central nervous system.

Ten studies have been identified which investigated the effect of Resv on blood glucose levels, using genetically obese mice or rats, dietary induced obesity or chemically induced diabetes by STZ or alloxan [21], [73], [83], [85], [94], [97]–[101]. Only one study [101], performed on rabbits with alloxan-induced diabetes, didn't find a reduced blood glucose level following exposure to Resv for 5 days to 2 months.

#### Neuroprotective action of resveratrol

Several animal studies have indicated that Resv has a neuroprotective effect (Table S4). In total, 12 studies test the effect of resveratrol after a single exposure and 28 studies investigated the effect of resveratrol af subchronic/chronic exposure. This effect of Resv has been documented in various animal models including rabbits [102], mice or rats and using different end points, such as reduced lipid peroxidation and neurological cell destruction [100], [103]–[107], attenuation of induced lesion areas [82], [108]–[115], induced tolerance to brain injury [116], reduced frequency of seizures [117], impairment of motor coordination [102], [104], [118]–[124] and enhancement of learning [125]–[127]. Only very few of these experiments found no or a marginal effect of Resv. A significant number of these studies use acute doses of Resv, ranging 5–100 mg/kg using one dose or exposure to the animals for Resv up to 1 week. Thirteen studies unraveling the neuroprotective effect of Resv expose to the experimental animals for more than 3 weeks: 10–50 mg Resv/kg bw/day for 3–6 weeks [100], [104], [105], [126], [128], [129], 100–300 mg Resv/kg bw/day for 3–6 weeks [107], [111], [122], 10–40 mg/kg bw/day for 10 weeks [130], or three weeks exposure of 2.5 µg Resv (by intra-cerebroventricular injection) every 2–3 day for 3 weeks [125].

#### Modulation of inflammation by resveratrol

Inflammatory response is a well known mechanism of the diseases described above such as cancer, coronary-heart disease, diabetes and neurodegeneration. Resveratrol is shown to modulate the inflammatory response induced by various stimuli. Fourteen studies have investigated the effect of exposure of one week or more to Resv on various inflammatory markers in rats. The same number of studies has been identified using mice as an experimental model. Generally, Resv in nearly all models counteracted the increased levels of pro inflammatory biochemical markers, such as TNFα, IL-1β, IL-6 in nearly all models. Beside these cytokines, MCP-1, COX-2 and iNOS was most often found to be down-regulated by Resv when stimulated by the pro-inflammatory treatment. The estimates of inflammation were often performed as a part of a study with another aim, i.e. testing a chemopreventive effect, or the effect of Resv on diabetes or cardiovascular disease. Therefore, different inducers of the inflammatory status have been used; Resv reduced inflammation in several models such as obesity-induced [73], in diabetic mice [131] or chemically induced diabetes [124], [132], [133], but also dextran sulfate sodium induced colon colitis [55], [134]–[138]. Other models were using induced hypertension [72], [139], chemicals causing tissue injury (and act as carcinogen) in liver [140], lung [141], [142] and colon [143]–[145] and showed decreased levels of inflammatory markers (Table S5).

Besides the animal experiments described above which focused on chronic or near-chronic exposures, a long list of papers exist that analyze the effect of Resv after an acute exposure on biomarkers relevant for prevention of cancer, coronary-heart disease and diabetes. These articles are not included in the present review.

The working group concluded (Table 4):

- There is sufficient evidence for a chemopreventive effect of resveratrol on the development of skin cancer in mice. There are promising results on the prevention of colon cancer in animals. The effects of resveratrol on other cancer types besides skin cancer need to be investigated more in detail prior to recommending clinical trials.
- There is sufficient evidence to suggest that resveratrol reduces the incidence of hypertension, heart failure, ischemia heart disease in experimental animal models.
- There is sufficient evidence to suggest that resveratrol improves insulin sensitivity, reduces blood glucose levels, and reduces high fat diet-induced obesity in rodents.
- Resveratrol showed neuroprotective effects in experimental animal models of injury or degeneration.
- Resveratrol is well tolerated in rats and no toxicological effects are observed up to 700–1000 mg/kg bw/day.

### Which relevant (overall) mechanisms of action of resveratrol have been documented?

Without going too much into details, and without giving the references, several mechanisms of action of Resv are relevant in relation to its proposed enhancement of human health. A number of mechanisms are relevant for several of the diseases mentioned herein, whereas others are more specific. The working group has identified twelve such mechanisms, as listed in Table 5. They are closely related and it is not possible to focus solely on one mechanism without taking the other mechanisms into consideration. Further, the indicated mechanisms are identified in *in vitro*, in experimental animals or both.

Suppression of inflammation is a general mechanism relevant for prevention of cancer diseases, coronary heart diseases, diabetes and neurodegeneration, as indicated above. Similarly, modulation of the cellular redox status is closely related to several diseases and linked to the anti-inflammatory effect. Modulation of cell proliferation and apoptosis as well as modulation of angiogenesis, inhibition of metastasis and suppression of DNA damage are relevant especially in cancer diseases. Modulation of xenobiotic metabolism by Resv likely plays a significant role in cancer prevention but may also have an impact on metabolism of drugs used to treat the listed diseases. Modulation of mitochondrial activity seems crucial in obesity/diabetes but may also be relevant for understanding life extension as well as be related to the inhibition of cell proliferation. Suppression of adipogenesis and stimulation of adipocyte lipolysis by Resv is relevant when one consider the effect on obesity and diabetes. Relevant for neuroprotection by Resv is the modulation of glutamate metabolism. Resveratrol also stimulates osteogenesis which indicates an effect on bone biology. Estrogenic activity but also anti-estrogenic activity by Resv has been shown but the clinical significance of these observations is uncertain.

### Recommendations on relevant research to be performed in the near future

It clearly follows from the recommendations for the use of Resv that evidence for the effect of Resv in humans is lacking. Table 7 shows the research in the field of Resv considered of highest priority. These recommendations naturally do not give space for a full list of relevant research projects, but only the most relevant from the point of view of the scientific working group of the Resveratrol2010 conference. The clinical studies with focus on cancer prevention might have a high priority, and as the most clear results have been shown in animal models of cancer in skin and colon these targets are the most relevant points of initiation. Furthermore, investigation of the effect of Resv on metabolic disorders evaluated in preclinical studies in nonhuman primates also has a high priority. Relevant clinical studies should be done soon and address the preventive effect of Resv on coronary heart disease. However, long term studies in animal models are still needed in order to evaluate the chronic effect of Resv to identify a probable NOAEL in human. Since chronic low grade inflammation is reduced by resveratrol and is a general characteristic of several of the life style disease, the effect of Resv on inflammatory biomarkers should be investigated in the coming clinical studies.

One of the major challenges in the clinical studies investigating the preventive effect of e.g. Resv is to show the absence or reduced incidence of a specific disease end point. Such intervention studies should then be long term and will therefore be very expensive. To overcome these challenges, new biomarkers need to be identified, developed and verified to analyze the long term disease prevention (Table 7).

To make preclinical and clinical studies comparable, the working group suggests that a standard Resv formulation should be performed. Such standardized Resv formulation should be made based on very pure Resv preparations. The use of non-pure Resv samples for preclinical or clinical experiments makes their interpretation very difficult. On the other hand, elucidation of the combined effect of Resv together with other dietary or non-dietary compounds should be accelerated as combinatory effects may solve some of the possible draw backs of high doses of resveratrol.

Besides using a standardized Resv formulation for the preclinical and clinical studies a Resv reference should be prepared for analytical purposes, such as analyzing metabolite formation and in the investigation of the bioavailability of Resv.

The bioavailability of Resv is described to some extent but have to be analyzed further to find the biodistribution and the degradation *in vivo*, which is very relevant to forecast the biological effect of Resv in humans. Several Resv metabolites have been identified but the biological activity of these metabolites formed in humans and experimental animals needs to be elucidated fully.

Lastly, the combinatory effect of Resv with other bioactive compounds has only been studied in few cases in experimental animals or *in vitro*. These studies should be amplified, as the outcomes of the combinations are not only additive but also synergistic or even antagonistic. The effect of Resv on the metabolism of these compounds have to be addressed as well, e.g. regulation of Cytochrome P450 enzymes and activities.

### Conclusions

The scientific literature cannot yet justify recommendation for the chronic administration of resveratrol to human beings, as stated in Table 6. Humans are receiving resveratrol frequently in red wine, berries, peanuts etc. but these levels are low, less than 4 mg. Before having data from chronic exposure to higher levels of resveratrol or more chronic studies with experimental animals, intake of resveratrol at higher doses should be considered experimental.

In contrast to the lacking data of resveratrol in humans, the animal data are promising and indicate the need for further human clinical trials. Therefore, the working group from the Resveratrol 2010 conference recommends that these human trials will be initiated soon.

## Supporting Information

Table S1 **Overview of the effect of resveratrol on cancer development in experimental animals.** To identify papers investigating the cancer preventive potential of resveratrol, a literature search using the terms “resveratrol” in combination with “cancer”, “carcinogenesis” or “chemoprevention” up to September 2010 was performed. In total, 1191 papers were identified, but only 41 papers were found to investigate the effect of resveratrol in animal model systems.
(DOCX)

Table S2 **Overview of the effect of resveratrol on coronary heart disease models in experimental animals.** To identify papers investigating the effect of resveratrol on coronary heart diseases, a literature search using the terms “resveratrol” in combination with “hypertension”, “heart failure”, “myocardial infarct”, “cardiac arrest”, “ischemia heart disease”, “stroke” or “serum lipids” up to September 2010 was done. 118 papers were found, but only 26 papers showed data from non acute animal experiments.
(DOCX)

Table S3 **Overview of experiments focusing on the effect of resveratrol on obesity and diabetes in experimental animals.** In total, 218 articles were identified, but only 19 animal studies were found to investigate the non-acute effects of resveratrol. The search terms “resveratrol” were used in combination with “metabolic syndrome”, “obesity”, “diabetes”, “insulin sensitivity”, “plasma glucose” or “visceral fat” in a literature search including papers published up to September 2010.
(DOCX)

Table S4 **Overview of the neuroprotective effect by resveratrol was in focus of 163 papers, of which 40 papers concerned animal with acute exposure and sub- and chronic exposure.** The search terms “resveratrol” was used in combination with “neuroprotection” or “neuroprotective” including papers published up to September 2010.
(DOCX)

Table S5 **Overview of the effect of resveratrol on inflammatory markers in experimental animals.** To identify papers investigating the effect of resveratrol on inflammatory markers, a literature search using the terms “resveratrol” in combination with “inflammation” or “inflammatory” was done, including papers published up to September 2010. In total, 559 papers were identified, whereas only 31 papers included sub-chronic and chronic exposures of resveratrol to experimental animals.
(DOCX)
