# Supplementary material for: What Is New for an Old Molecule? Systematic Review and Recommendations on the Use of Resveratrol
Source: PLoS One. 2011 Jun 16;6(6):e19881. doi: 10.1371/journal.pone.0019881 (PMC3116821; doi:10.1371/journal.pone.0019881)
Supplement: Table S1 — Overview of the effect of resveratrol on cancer development in experimental animals. To identify papers investigating the cancer preventive potential of resveratrol, a literature search using the terms “resveratrol” in combination with “cancer”, “carcinogenesis” or “chemoprevention” up to September 2010 was performed. In total, 1191 papers were identified, but only 41 papers were found to investigate the effect of resveratrol in animal model systems. (DOCX) [file pone.0019881.s001.docx]

What is new for an old Molecule? Systematic Review and Recommendations on the use of Resveratrol

Ole Vang, Nihal Ahmad, Clifton A. Baile, Joseph A. Baur, Karen Brown et al.

Supporting information:

| **Table S1:** Effect of resveratrol on cancer development in experimental animals | | | | | |
| --- | --- | --- | --- | --- | --- |
|  | | | | | |
| **Species / Strain** | **Carcinogen** | **Resveratrol dose** | **Duration** | **Effect** | **References** |
| Skin - Non-melanoma skin cancer | | | | | |
| Female C3H/HeN mice | DMBA, 400 nmol, 1x / week | 10 µmol/ mouse prior carcinogen treatment | 25 weeks | Incidence: ↓ | [1] |
| SENCAR mice | DMBA |  | 4 weeks | Hyperplasia: ↓ | [2] |
| Female ICR mice | DMBA, 390 nmol/ mouse, 1.7 nmol TPA twice a week after 1 week and rest of exp. | 8.5 pmol Resv topically daily, 1 week prior initiation | 20 weeks | Multiplicity: ↓  Incidence: ↓ | [3] |
| Female CD-1 mice | Topically, 200 µmol of DMBA + 5 mmol of TPA | 1 – 25 µmol Resv, twice / week | 18 weeks | Incidence: ↓ all conc. used | [4] |
| CD-1 mice | Topically, 200 nmol DMBA + 5 nmol of TPA twice a week | 1 – 25 µmol Resv, twice / week | 18 weeks | Incidence: ↓ all conc. used | [5] |
| Female Swiss mice | Topically, DMBA (5 μg/animal) | 5 – 10 nmol Resv, 1 hour before DMBA | 28 weeks | Incidence: ↓ all conc. used | [6] |
| Skin – Melanoma | | | | | |
| Male Wistar rats | ip injection with AH-130 Yoshida ascites hepatoma cells | ip injection of 1 mg Resv/ kg bw/ day | 7 days | Growth of injected cell: ↓ | [7] |
| Male Nu/Nu-nuRB mice |  | 0.0025 or 0.006% Resv in diet (~ 3 or 7.2 mg Resv/ kg bw/ day) | 28 weeks | Latency: →  Incidence: → | [8] |
| Female C57BL/6N mice | B16-BL6 melanoma cells | 50 mg/ kg Resv ip injection daily | 9 days | Growth of injected cell: ↓ | [9] |
| Breast | | | | | |
| Female CD-1 mice | Top. appli. of DMBA | Pre incub. with 0.01 – 100 µM Resv twice / week | 18 weeks | Incidence: ↓ all conc. used | [4] |
| FVB/N female mice |  | 1 mg/l in drinking water, from week 20 (~ 0.1mg Resv/ kg bw/ day) | 10 weeks | Latency: ↓ | [10] |
| Female BALB/c mice | Injected mammary 4T1 mammary carcinoma cells | 1, 3 or 5 mg Resv/kg, ip daily | 23 days | Tumor volume: →  Lung metastases: → | [11] |
| Female Swiss mice | Ehrlich ascites carcinoma cells from a spontaneous mammary cancer | 20 or 40 mg Resv/kg, ip daily | 20 days | Tumor size:↓ (40 mg/kg) | [12] |
| Female Sprague-Dawley rats | Single i.v. dose of NMU, 50 mg/kg | 10 and 100 mg/ kg bw/ day ig, 5 days/week, starting 1 week prior MNU | 120 days | Latency: ↓, Multiplicity: ↓  Incidence: → | [13] |
| Female Sprague-Dawley CD rats | 60 mg DMBA /kg | Resv (1 g/kg diet) for full life span (~ 50 mg Resv/ kg bw/ day) | 18 weeks | Latency: ↓, Multiplicity: ↓  Incidence: → | [14] |
| Female Sprague-Dawley rats | Day 52 of age: 10 mg DMBA | Day 45 of age and the rest of study: 0.5 mg Resv / kg bw (in the diet) | 120 days after DMBA treatment | Multiplicity: ↓  Incidence: ↓ | [15] |
| Female Sprague–Dawley rats | Single i.v. dose of NMU, 50 mg/kg (on day 49) | sc inject. Resv (10 or  100 mg/ kg) for five days | 40 weeks | Incidence: ↑ | [16] |
| Liver cancer | | | | | |
| Balb/C mice | Injected mouse hepatocellular carcinoma  cells H22 | ip injected with Resv: 500, 1000 or 1500 mg/ kg | 10 days | Growth of H22 *in vivo:* ↓ , all conc. Used | [17] |
| Balb/c mice | Transplanted hepatoma cells | ip injected with Resv: 5, 10 or 15 mg/ kg | 10 days | Tumor size: ↓ , all conc. used | [18] |
| C57BL/6J mice | Intrasplenic injection B16 melanoma cells | Oral: 20 mg/kg 2 times/ day or 23 mg/L in drinking water | 10 days | Metastasis: ↓ | [19] |
| Sprague–Dawley rats | DENA, 200mg/kg, ip +  phenobarbital (0.05%) in  drinking water | Resv in diet equiv. to 50, 100 or 300 mg/kg bw /day, start 4 weeks prior initiat. | 4 + 20 weeks | Incidence: ↓ all conc. used  Multiplicity: ↓ all conc. used | [20] |
| Esophagus | | | | | |
| Male Sprague-Dawley rats | Esophagoduodenal anastomosis | ip injection with 7 mg Resv / kg | 5 months | Esophagitis: ↓  Incidence of intestinal metaplasia: →  Incidence of carcinoma: → | [21] |
| Gastric cancer | | | | | |
| Female Balb/C nude mice | sc injection of human primary gastric  cancer cells | 500, 1000 or 1500 mg/kg Resv, injected beside tumor, 6x over 2 days | 2 days | Tumor volume:↓ (all doses) | [22] |
| Male NMRI mice | sc implanted with the MAC16 tumor cells | After 10-12 days: 1 mg Resv/ kg ip injection daily | 3 days | Tumor volume:↓ | [23] |
| Colorectal cancer | | | | | |
| Male Wistar rats | 20 mg DMH /kg, once a week for 15 weeks | 8 mg Resv/ kg / day, either simultaneously with DMH, after DMH treatment or in the entire period | 15 / 30 weeks | ACF: entire treatm. Period: ↓  Adenoma / Adenocarcinoma: ↓  Incidence:↓, Multiplicity: ↓ | [24–26] |
| Male F344 rats | 15 mg AOM/ kg 2x 1 week apart | 10 days prior to carcinog. treatment: 200 µg / kg bw/ day in drinking water | 100 days | ACF incidence: ↓  ACF multiplicity: ↓ | [27] |
| CF-1 mice | ip injection of AOM (5 mg/kg body weight) | 20 mg/kg diet (~ 2.4 mg Resv/ kg bw/ day) | 5 weeks | Total ACF: ↓  ACF distal part: ↓  ACF multiplicity: → | [28] |
| Male C57BL/6J ^ApcMin/+^ mice |  | 0.05% or 0.2% Resv in the diet (~ 60 and 240 mg Resv/ kg bw/ day). | 3 weeks | Adenomas: ↓ (0.2 % Resv) | [29] |
| Male C57BL/6J ^ApcMin/+^ mice |  | 0.01% Resv in drinking water (~ 12 mg Resv/ kg) | 7 weeks | Small intestine adenomas: ↓  Colon adenomas: → | [30] |
| Male C57BL/6J ^ApcMin/+^ mice |  | Dietary Resv correspond. to 0, 4, 20 and 90 mg/ kg bw/ day | 7 weeks | Tumor load: → | [31] |
| Male and female C57BL/6 mice | 10 mg AOM/kg bw  Water containing 1% DSS | 300 ppm Resv in diet (~ 48 mg/ kg bw/ day) | 62 days | Tumor incidence: ↓  Multiplicity: ↓ | [32] |
| Prostate | | | | | |
| Male Sprague–Dawley rats (SV-40 Tag) |  | 250 mg Resv/ kg in diet (~ 15 mg/ kg bw/ day) | 30 weeks | Incidence: ↓ | [33] |
| Male heterozygous TRAP rats |  | 50, 100 or 200 µg Resv/ ml in drinking water (~ 5, 10 or 20 mg/ kg bw/ day) | 7 weeks | Neoplastic lesions:↓ | [34] |
| Male PTEN-KO  mice |  | 50 mg / kg / by oral gavages, 3 times a week | 7 weeks | Adenoma incidence :↓ | [35] |
| Male athymic nude  BALB/cAnNCr-nu/nu mice | sc injection of LNCaP cells | 50 or 100 mg / kg diet, starting 2 weeks before inoculation of cells water (~ 6 or 12 mg/ kg bw/ day) | 9 weeks | tumor growth:↓ | [36] |
| Lung | | | | | |
| Female A/JOlaHsd mice | 80 or 300mg B[a]P /kg bw (gastric tube) once a week for 8 weeks | 0.4% Resv in diet  (~ 280 mg/ kg bw/ day) | 6 months | Incidence: →, Multiplicity: → | [37] |
| Female A/J mice | Weekly gavages doses of BaP + NNK (3 µmol of each) for eight weeks | 500 ppm in diet, starting 1  week after the final dose of BaP and NNK (~ 60 mg/ kg bw/ day) | 26 weeks, 18 weeks with Resv | Incidence: →, Multiplicity: → | [38] |
| Female C57BL/6N mice | B16-BL6 melanoma cells | 50 mg/ kg Resv ip injection daily | 9 days | Lung colonization: → | [9] |
| Female C57BL/6  mice | sc injection of Lewis lung carcinoma cells | Resv (0.6, 2.5 or 10 mg/ kg) ip once daily | 22 days | 2.5 or 10 mg/kg Resv:  Tumor weight: ↓  Tumor volume: ↓ | [39] |
| Neuroblastoma | | | | | |
| Male A/J mice | sc injection with neuro-2a cells | 40 mg Resv /kg / day | 28 days | Tumor volume: ↓ | [40] |
| Leukemia | | | | | |
| BALB/c mice | ip injection with L1210 cells | ig administration 12.5, 25, 50 mg/ kg /day | 3 weeks | Life span of tumor-bearing mice (dose-dependently): ↑ | [41] |
| ip: intraperitoneally; ig: intragastically; sc: subcutaneous; bw: body weight  AOM: Azoxymethane; B[a]P: Benzo[a]pyrene; DENA: Diethylnitrosamine; DMBA: 7,12-dimethylbenzanthracene; DMH: 1,2-dimethylhydrazine; DSS: dextran sulfate sodium; NNK: 4-(methylnitrosamino)-1-(3-pyridyl)-1-butanone; TPA: 12-0-tetradecanoylphorbol-13-acetate;  ACF: aberrant crypt foci;  Effect is indicated by ↓: reduction; ↑: enhancement; →: no effect. | | | | | |

**References**

1. Yusuf N, Nasti TH, Meleth S, Elmets CA (2009) Resveratrol enhances cell-mediated immune response to DMBA through TLR4 and prevents DMBA induced cutaneous carcinogenesis. Mol Carcinogen 48: 713-723.

2. Kowalczyk MC, Kowalczyk P, Tolstykh O, Hanausek M, Walaszek Z et al. (2010) Synergistic effects of combined phytochemicals and skin cancer prevention in SENCAR mice. Cancer Prev Res 3: 170-178.

3. Kapadia GJ, Azuine MA, Tokuda H, Takasaki M, Mukainaka T et al. (2002) Chemopreventive effect of resveratrol, sesamol, sesame oil and sunflower oil in the Epstein-Barr virus early antigen activation assay and the mouse skin two-stage carcinogenesis. Pharmacol Res 45: 499-505.

4. Jang M, Cai L, Udeani GO, Slowing KV, Thomas CF et al. (1997) Cancer chemopreventive activity of resveratrol, a natural product derived from grapes. Science 275: 218-220.

5. Soleas GJ, Grass L, Josephy PD, Goldberg DM, Diamandis EP (2002) A comparison of the anticarcinogenic properties of four red wine polyphenols. Clin Biochem 35: 119-124.

6. Roy P, Kalra N, Prasad S, George J, Shukla Y (2009) Chemopreventive Potential of Resveratrol in Mouse Skin Tumors Through Regulation of Mitochondrial and PI3K/AKT Signaling Pathways. Pharm Res 26: 211-217.

7. Carbo N, Costelli P, Baccino FM, Lopez-Soriano FJ, Argilos JM (1999) Resveratrol, a natural product present in wine, decreases tumour growth in a rat tumour model. Biochem Biophys Res Commun 254: 739-743.

8. Niles RM, Cook CP, Meadows GG, Fu YM, McLaughlin JL et al. (2006) Resveratrol is rapidly metabolized in athymic (Nu/Nu) mice and does not inhibit human melanoma xenograft tumor growth. J Nutr 136: 2542-2546.

9. Caltagirone S, Rossi C, Poggi A, Ranelletti FO, Natali PG et al. (2000) Flavonoids apigenin and quercetin inhibit melanoma growth and metastatic potential. Int J Cancer 87: 595-600.

10. Provinciali M, Re F, Donnini A, Orlando F, Bartozzi B et al. (2005) Effect of Resveratrol on the development of spontaneous mammary tumors in HER-2/neu transgenic mice. Int J Cancer 115: 36-45.

11. Bove K, Lincoln DW, Tsan MF (2002) Effect of resveratrol on growth of 4T1 breast cancer cells in vitro and in vivo. Biochem Biophys Res Commun 291: 1001-1005.

12. El-Mowafy AM, El-Mesery ME, Salem HA, Al-Gayyar MM, Darweish MM (2010) Prominent chemopreventive and chemoenhancing effects for resveratrol: unraveling molecular targets and the role of C-reactive protein. Chemotherapy 56: 60-65.

13. Bhat KPL, Lantvit D, Christov K, Mehta RG, Moon RC et al. (2001) Estrogenic and antiestrogenic properties of resveratrol in mammary tumor models. Cancer Res 61: 7456-7463.

14. Whitsett TG, Jr., Carpenter DM, Lamartiniere CA (2006) Resveratrol, but not EGCG, in the diet suppresses DMBA-induced mammary cancer in rats. J Carcinog 5: 15.

15. Banerjee S, Bueso-Ramos C, Aggarwal BB (2002) Suppression of 7,12-dimethylbenz(a)anthracene-induced mammary carcinogenesis in rats by resveratrol: Role of nuclear factor-kappa B, cyclooxygenase 2, and matrix metalloprotease 9. Cancer Res 62: 4945-4954.

16. Sato M, Pei RJ, Yuri T, Danbara N, Nakane Y et al. (2003) Prepubertal resveratrol exposure accelerates N-methyl-N-nitrosourea-induced mammary carcinoma in female Sprague-Dawley rats. Cancer Lett 202: 137-145.

17. Liu HS, Pan CE, Yang W, Liu XM (2003) Antitumor and immunomodulatory activity of resveratrol on experimentally implanted tumor of H22 in Balb/c mice. World J Gastroenterol 9: 1474-1476.

18. Yu L, Sun ZJ, Wu SL, Pan CE (2003) Effect of resveratrol on cell cycle proteins in murine transplantable liver cancer. World J Gastroenterol 9: 2341-2343.

19. Asensi M, Medina I, Ortega A, Carretero J, Bano MC et al. (2002) Inhibition of cancer growth by resveratrol is related to its low bioavailability. Free Radic Biol Med 33: 387-398.

20. Bishayee A, Dhir N (2009) Resveratrol-mediated chemoprevention of diethylnitrosamine-initiated hepatocarcinogenesis: inhibition of cell proliferation and induction of apoptosis. Chem Biol Interact 179: 131-144.

21. Woodall CE, Li Y, Liu QH, Wo J, Martin RC (2009) Chemoprevention of metaplasia initiation and carcinogenic progression to esophageal adenocarcinoma by resveratrol supplementation. Anti Cancer Drug 20: 437-443.

22. Zhou HB, Chen JJ, Wang WX, Cai JT, Du Q (2005) Anticancer activity of resveratrol on implanted human primary gastric carcinoma cells in nude mice. World J Gastroenterol 11: 280-284.

23. Wyke SM, Russell ST, Tisdale MJ (2004) Induction of proteasome expression in skeletal muscle is attenuated by inhibitors of NF-kappaB activation. Br J Cancer 91: 1742-1750.

24. Sengottuvelan M, Senthilkumar R, Nalini N (2006) Modulatory influence of dietary resveratrol during different phases of 1,2-dimethylhydrazine induced mucosal lipid-peroxidation, antioxidant status and aberrant crypt foci development in rat colon carcinogenesis. Biochimica et Biophysica Acta-General Subjects 1760: 1175-1183.

25. Sengottuvelan M, Nalini N (2006) Dietary supplementation of resveratrol suppresses colonic tumour incidence in 1,2-dimethylhydrazine-treated rats by modulating biotransforming enzymes and aberrant crypt foci development. Br J Nutr 96: 145-153.

26. Sengottuvelan M, Viswanathan P, Nalini N (2006) Chemopreventive effect of trans-resveratrol--a phytoalexin against colonic aberrant crypt foci and cell proliferation in 1,2-dimethylhydrazine induced colon carcinogenesis. Carcinogenesis 27: 1038-1046.

27. Tessitore L, Davit A, Sarotto I, Caderni G (2000) Resveratrol depresses the growth of colorectal aberrant crypt foci by affecting bax and p21(CIP) expression. Carcinogenesis 21: 1619-1622.

28. Kineman BD, Au A, Paiva NL, Kaiser MS, Brummer EC et al. (2007) Transgenic alfalfa that accumulates piceid (Trans-Resveratrol-3-O-Beta-D-glucopyranoside) requires the presence of beta-glucosidase to inhibit the formation of aberrant crypt foci in the colon of CF-1 mice. Nutr Cancer 58: 66-74.

29. Sale S, Tunstall RG, Ruparelia KC, Potter GA, Steward WP et al. (2005) Comparison of the effects of the chemopreventive agent resveratrol and its synthetic analog trans 3,4,5,4 '-tetramethoxystilbene (DMU-212) on adenoma development in the Apc(Min+) mouse and cyclooxygenase-2 in human-derived colon cancer cells. Int J Cancer 115: 194-201.

30. Schneider Y, Duranton B, Gosse F, Schleiffer R, Seiler N et al. (2001) Resveratrol inhibits intestinal tumorigenesis and modulates host-defense-related gene expression in an animal model of human familial adenomatous polyposis. Nutr Cancer 39: 102-107.

31. Ziegler CC, Rainwater L, Whelan J, McEntee MF (2004) Dietary resveratrol does not affect intestinal tumorigenesis in Apc(Min/+) mice. J Nutr 134: 5-10.

32. Cui X, Jin Y, Hofseth AB, Pena E, Habiger J et al. (2010) Resveratrol suppresses colitis and colon cancer associated with colitis. Cancer Prev Res 3: 549-559.

33. Harper CE, Cook LM, Patel BB, Wang J, Eltoum IA et al. (2009) Genistein and resveratrol, alone and in combination, suppress prostate cancer in SV-40 tag rats. Prostate 69: 1668-1682.

34. Seeni A, Takahashi S, Takeshita K, Tang M, Sugiura S et al. (2008) Suppression of prostate cancer growth by resveratrol in the transgenic rat for adenocarcinoma of prostate (TRAP) model. Asian Pac J Cancer Prev 9: 7-14.

35. Narayanan NK, Nargi D, Randolph C, Narayanan BA (2009) Liposome encapsulation of curcumin and resveratrol in combination reduces prostate cancer incidence in PTEN knockout mice. Int J Cancer 125: 1-8.

36. Wang TT, Hudson TS, Wang TC, Remsberg CM, Davies NM et al. (2008) Differential effects of resveratrol on androgen-responsive LNCaP human prostate cancer cells in vitro and in vivo. Carcinogenesis 29: 2001-2010.

37. Berge G, Ovrebo S, Eilertsen E, Haugen A, Mollerup S (2004) Analysis of resveratrol as a lung cancer chemopreventive agent in A/J mice exposed to benzo[alpha] pyrene. Br J Cancer 91: 1380-1383.

38. Hecht SS, Kenney PM, Wang M, Trushin N, Agarwal S et al (1999) Evaluation of butylated hydroxyanisole, myo-inositol, curcumin, esculetin, resveratrol and lycopene as inhibitors of benzo[a]pyrene plus 4-(methylnitrosamino)-1-(3-pyridyl)-1-butanone-induced lung tumorigenesis in A/J mice. Cancer Lett 137: 123-130.

39. Kimura Y, Okuda H (2001) Resveratrol isolated from Polygonum cuspidatum root prevents tumor growth and metastasis to lung and tumor-induced neovascularization in Lewis lung carcinoma-bearing mice. J Nutr 131: 1844-1849.

40. Chen Y, Tseng SH, Lai HS, Chen WJ (2004) Resveratrol-induced cellular apoptosis and cell cycle arrest in neuroblastoma cells and antitumor effects on neuroblastoma in mice. Surgery 136: 57-66.

41. Li T, Fan GX, Wang W, Li T, Yuan YK (2007) Resveratrol induces apoptosis, influences IL-6 and exerts immunomodulatory effect on mouse lymphocytic leukemia both in vitro and in vivo. Int Immunopharmacol 7: 1221-1231.
