# Supplementary material for: What Is New for an Old Molecule? Systematic Review and Recommendations on the Use of Resveratrol
Source: PLoS One. 2011 Jun 16;6(6):e19881. doi: 10.1371/journal.pone.0019881 (PMC3116821; doi:10.1371/journal.pone.0019881)
Supplement: Table S2 — Overview of the effect of resveratrol on coronary heart disease models in experimental animals. To identify papers investigating the effect of resveratrol on coronary heart diseases, a literature search using the terms “resveratrol” in combination with “hypertension”, “heart failure”, “myocardial infarct”, “cardiac arrest”, “ischemia heart disease”, “stroke” or “serum lipids” up to September 2010 was done. 118 papers were found, but only 26 papers showed data from non acute animal experiments. (DOCX) [file pone.0019881.s002.docx]

What is new for an old Molecule? Systematic Review and Recommendations on the use of Resveratrol

Ole Vang, Nihal Ahmad, Clifton A. Baile, Joseph A. Baur, Karen Brown et al.

Supporting information:

| **Table S2:** Effect of resveratrol on coronary heart disease models in experimental animals | | | | | |
| --- | --- | --- | --- | --- | --- |
|  | | | | | |
| **Species / Strain** | **Model** | **Resveratrol dose** | **Duration** | **Effect** | **References** |
| Hypertension | | | | | |
| Wistar-Kyoto rats (WKY) and spontaneously hypertensive rat (SHR) | Spontaneously hypertensive rat | 2.5 mg Resv/ kg bw/ day | 10 weeks | SHR: Vascular compliance ↓; wall component stiffness →  WKY: Vascular compliance ↑; wall component stiffness ↓  SHR: Elevated blood pressure → | [1] |
| Wistar-Kyoto rats and spontaneously hypertensive rat (SHR) | Spontaneously hypertensive rat | 2.5 mg Resv/ kg bw/ day | 10 weeks | SHR: Increased systolic blood pressure →  Development of concentric hypertrophy ↓  Systolic and diastolic dysfunction ↓ | [2] |
| Hypertensive transgenic rats, controls: normotensive Sprague – Dawley | Rats transgenic with human renin and angiotensinogen genes | 800 mg Resv /kg bw/ day by gavages | 4 weeks | Survival rate of dTGR ↑  Blood pressure ↓  Cardiac hypertrophy ↓ | [3] |
| Male Wistar rats | 25 mg DOCA every 4th day to uninephrectomized rats | 1 mg Resv/ kg by  oral gavages, starting 4 days before surgery until end of experiment | 4 weeks | Increased systolic blood pressure ↓  Left ventricular wet weight ↓  Left ventricular wall thickness ↓  Diastolic stiffness ↓  Cardiac contractility ↑  Prolonged action potential ↓ | [4] |
| Male Sprague-Dawley rats | A single injection of  MCT (50 mg/kg, sc) | 10 and 30 mg Resv/ kg bw, ig, twice daily | 21 days | Survival rate after MCT injection ↑  Right ventricular (RV) wall thickness ↓  RV systolic pressure ↓  Pulmonary arterial acceleration time ↑  RV hypertrophy ↓ | [5] |
| Male Sprague-Dawley rats | MCT (60 mg/kg sc) | Resv (25 mg/kg per day, po, from day 1 post MCT  in the drinking water | 14 / 21 days | RV systolic pressure ↓  and pulmonary arterial remodeling ↓  normalization of vessel morphology | [6] |
| Female Sprague-Dawley | High-fat diet (42% fat) | 20 mg/kg bw/day Resv | 8 weeks | Increased blood pressure ↓ | [7] |
| Lean /Obese Zucker rats |  | 10 mg Resv/kg / day orally by gavages | 8 weeks | Obese: Systolic blood pressure ↓  Lean: Systolic blood pressure → | [8] |
| Sprague Dawley rats | Fed fructose (60%) -enriched food | 2.1 mg Resv/ kg bw/ day by gavages | 6 weeks | Fructose induced hypertension → | [9] |
| Male Sprague Dawley rats | Fructose (10% in drinking water) | 10 mg Resv/ kg bw/ day by gavages | 45 days | Fructose induced stolic blood pressure ↓  Cardiac hypertrophy ↓ | [10] |
| C57/B6 mice | 490 ng Ang II/ min/ kg, ip | Resv in water at 0.1 mg/ml (~10 mg / kg bw/ day) | 2 / 4 weeks | Ang II induced blood pressure ↓ | [11] |
| Heart failure / myocardial infarction / cardiac arrest | | | | | |
| Male Sprague–Dawley  rats | Myocardial infarction (MI) operated | 5 mg/kg bw/day po | 4 weeks, starting 1 week before MI | MI-induced ventricular tachycardia ↓  MI induced ventricular fibrillation ↓  Myocardial infarct size ↓  Mortality ↓ | [12] |
| Sprague–Dawley rats | MI operated | 0.1 or 1 mg Resv/ kg bw/ day, one daily ip injection | 4 weeks | Myocardial infarct size ↓  Fractional shortening of the left ventricle ↑  Ameliorated left ventricular dilatation  Left ventricular end-diastolic pressure ↓ | [13] |
| Male Sprague-Dawley rats | MI operated | 17 mg/ kg bw/ day | 3 months | Myocardial infarct size →  MI-induced left-ventricular →  Left-atrial dilatation →  Reduction in left-ventricular fractional shortening → | [14] |
| Male Sprague-Dawley rats | MI operated | 10 mg Resv/ kg bw/ day | 1 week | Left ventricular function ↑ | [15] |
| Sprague-Dawley rats | Aortocaval shunt to  create volume overload and Abdominal aortic banding surgeries to create pressure overload | 2.5 mg Resv/ kg bw/ day  - 2 d post surgery  -14 d post surgery | for 26 days  for 14 days | The development of abnormalities in cardiac structure and function ↓ | [16] |
| Sprague–Dawley rats | A single intravenous injection of STZ (65 mg/kg) for 2 weeks | 0.1 or 1 mg Resv/ kg + insulin  once a day | 5 days | Acute myocardial ischemia/reperfusion ↓ | [17] |
| Zucker obese rats | 10% glucose solution ad libitum | 5 mg Resv/ kg bw/ day | 2 weeks | Incidence of ventricular fibrillation ↓  Myocardial infarct size↓ | [18] |
| Sprague Dawley rats | 65 mg STZ/kg bw/ day | 2.5 mg Resv/ kg bw/ day | 15 days | myocardial infarct size ↓ | [19] |
| Ischemia heart disease | | | | | |
| Sprague-Dawley rats | 2% cholesterol diet for 8 weeks.  ischemia / reperfusion | 20mg Resv/ kg bw/ day | 2 weeks | Left ventricular functional recovery ↑  Capillary density ↑ | [20] |
| Male Sprague–Dawley rats | Middle cerebral artery (MCA) occlusions | 2x10^-3^, 2x10^-4^, 1x10^-4^, 2x10^-5^  mg Resv/ kg bw | 4 hr | Infarct area ↓ | [21] |
| Yorkshire swine | Hypercholesterolemic diet (HCC) | 100 mg Resv/ kg bw/ day, po | 7 weeks | Total cholesterol↓  HCC reduced inferolateral function ↑  Tissue blood flow during stress ↑ | [22] |
| Stroke | | | | | |
| Male Wistar rats | Focal ischemia by MCA occlusion intraluminal thread | resveratrol 20 mg/kg bw ip | 21 days | Resv prevented motor impairment after focal cerebral ischemia  Enhanced locomotion and neurological score by resveratrol | [23] |
| Serum lipids | | | | | |
| Male Wistar rats | Diabetes-induced by single ip injection of 60 mg/ kg STZ – 1 week | po dosage  of 10 mg Resv/ kg bw/ day | 4 weeks / 8 weeks | Serum triglycerides (4 & 8 weeks) ↓  HDL-cholesterol (4 & 8 weeks) ↑  LDL-cholesterol (4 & 8 weeks) ↓ | [24] |
| Apolipoprotein E KO mice |  | P183/1-mixture: 27% Resv, 1.37 % caffeic acid and 8.35% cathechin | 8 weeks | Morphometric analysis: atherosclerosis ↓ | [25] |
| Ang II: Angiotensin II; DOCA: Deoxycorticosterone acetate; HCC: Hypercholesterolemic diet; MCT: monocrotalin; MCA: middle cerebral artery; MI: Myocardial infarction; RV: Right ventricular; SHR: spontaneously hypertensive rat; STZ: Streptozotocin;  Bw: body weight; ig: intragastrically; ip: intraperitoneally; po: per oral;  Effect are indicated by ↓: reduction; ↑: enhancement; →: no effect. | | | | | |

**References**

1. Behbahani J, Thandapilly SJ, Louis XL, Huang Y, Shao Z et al. (2010) Resveratrol and small artery compliance and remodeling in the spontaneously hypertensive rat. Am J Hypertens 23: 1273-1278.

2. Thandapilly SJ, Wojciechowski P, Behbahani J, Louis XL, Yu L et al. (2010) Resveratrol prevents the development of pathological cardiac hypertrophy and contractile dysfunction in the SHR without lowering blood pressure. Am J Hypertens 23: 192-196.

3. Biala A, Tauriainen E, Siltanen A, Shi J, Merasto S et al. (2010) Resveratrol induces mitochondrial biogenesis and ameliorates Ang II-induced cardiac remodeling in transgenic rats harboring human renin and angiotensinogen genes. Blood Press 19: 196-205.

4. Chan V, Fenning A, Iyer A, Hoey A, Brown L (2011) Resveratrol improves cardiovascular function in DOCA-salt hypertensive rats. Curr Pharm Biotechnol 12: 429-436.

5. Yang DL, Zhang HG, Xu YL, Gao YH, Yang XJ et al. (2010) Resveratrol inhibits right ventricular hypertrophy induced by monocrotaline in rats. Clin Exp Pharmacol Physiol 37: 150-155.

6. Csiszar A, Labinskyy N, Olson S, Pinto JT, Gupte S et al. (2009) Resveratrol prevents monocrotaline-induced pulmonary hypertension in rats. Hypertension 54: 668-675.

7. Aubin MC, Lajoie C, Clement R, Gosselin H, Calderone A et al. (2008) Female rats fed a high-fat diet were associated with vascular dysfunction and cardiac fibrosis in the absence of overt obesity and hyperlipidemia: therapeutic potential of resveratrol. J Pharmacol Exp Ther 325: 961-968.

8. Rivera L, Moron R, Zarzuelo A, Galisteo M (2009) Long-term resveratrol administration reduces metabolic disturbances and lowers blood pressure in obese Zucker rats. Biochem Pharmacol 77: 1053-1063.

9. Sutra T, Oiry C, zay-Milhau J, Youl E, Magous R et al. (2008) Preventive effects of nutritional doses of polyphenolic molecules on cardiac fibrosis associated with metabolic syndrome: Involvement of osteopontin and oxidative stress. J Agric Food Chem 56: 11683-11687.

10. Miatello R, Vazquez M, Renna N, Cruzado M, Zumino AP et al. (2005) Chronic administration of resveratrol prevents biochemical cardiovascular changes in fructose-fed rats. Am J Hypertens 18: 864-870.

11. Inanaga K, Ichiki T, Matsuura H, Miyazaki R, Hashimoto T et al. (2009) Resveratrol attenuates angiotensin II-induced interleukin-6 expression and perivascular fibrosis. Hypertens Res 32: 466-471.

12. Chen YR, Yi FF, Li XY, Wang CY, Chen L et al. (2008) Resveratrol attenuates ventricular arrhythmias and improves the long-term survival in rats with myocardial infarction. Cardiovasc Drugs Ther 22: 479-485.

13. Lin JF, Lin SM, Chih CL, Nien MW, Su HH et al. (2008) Resveratrol reduces infarct size and improves ventricular function after myocardial ischemia in rats. Life Sci 83: 313-317.

14. Burstein B, Maguy A, Clement R, Gosselin H, Poulin F et al. (2007) Effects of resveratrol (trans-3,5,4'-trihydroxystilbene) treatment on cardiac remodeling following myocardial infarction. J Pharmacol Exp Ther 323: 916-923.

15. Fukuda S, Kaga S, Zhan L, Bagchi D, Das DK et al. (2006) Resveratrol ameliorates myocardial damage by inducing vascular endothelial growth factor-angiogenesis and tyrosine kinase receptor Flk-1. Cell Biochem Biophys 44: 43-49.

16. Wojciechowski P, Juric D, Louis XL, Thandapilly SJ, Yu L et al. (2010) Resveratrol arrests and regresses the development of pressure overload - but not volume overload-induced cardiac hypertrophy in rats. J Nutr 140: 962-968.

17. Huang JP, Huang SS, Deng JY, Chang CC, Day YJ et al. (2010) Insulin and resveratrol act synergistically, preventing cardiac dysfunction in diabetes, but the advantage of resveratrol in diabetics with acute heart attack is antagonized by insulin. Free Radic Biol Med 49: 1710-1721.

18. Lekli I, Szabo G, Juhasz B, Das S, Das M et al. (2007) Protective mechanisms of resveratrol against ischemia/reperfusion-induced damage in hearts obtained from Zucker obese rats: the role of GLUT-4 and endothelin. Am J Physiol Heart Circ Physiol 294: H859-H866.

19. Thirunavukkarasu M, Penumathsa SV, Koneru S, Juhasz B, Zhan L et al. (2007) Resveratrol alleviates cardiac dysfunction in streptozotocin-induced diabetes: Role of nitric oxide, thioredoxin, and heme oxygenase. Free Radic Biol Med 43: 720-729.

20. Penumathsa SV, Thirunavukkarasu M, Koneru S, Juhasz B, Zhan L et al. (2007) Statin and resveratrol in combination induces cardioprotection against myocardial infarction in hypercholesterolemic rat. J Mol Cell Cardiol 42: 508-516.

21. Saleh MC, Connell BJ, Saleh TM (2010) Resveratrol preconditioning induces cellular stress proteins and is mediated via NMDA and estrogen receptors. Neuroscience 166: 445-454.

22. Robich MP, Osipov RM, Nezafat R, Feng J, Clements RT et al. (2010) Resveratrol improves myocardial perfusion in a swine model of hypercholesterolemia and chronic myocardial ischemia. Circulation 122: S142-S149.

23. Sinha K, Chaudhary G, Gupta YK (2002) Protective effect of resveratrol against oxidative stress in middle cerebral artery occlusion model of stroke in rats. Life Sci 71: 655-665.

24. Roghani M, Baluchnejadmojarad T (2010) Mechanisms underlying vascular effect of chronic resveratrol in streptozotocin-diabetic rats. Phytother Res S148-S154.

25. Norata GD, Marchesi P, Passamonti S, Pirillo A, Violi F et al. (2007) Anti-inflammatory and anti-atherogenic effects of cathechin, caffeic acid and trans-resveratrol in apolipoprotein E deficient mice. Atherosclerosis 191: 265-271.
