# Supplementary material for: What Is New for an Old Molecule? Systematic Review and Recommendations on the Use of Resveratrol
Source: PLoS One. 2011 Jun 16;6(6):e19881. doi: 10.1371/journal.pone.0019881 (PMC3116821; doi:10.1371/journal.pone.0019881)
Supplement: Table S3 — Overview of experiments focusing on the effect of resveratrol on obesity and diabetes in experimental animals. In total, 218 articles were identified, but only 19 animal studies were found to investigate the non-acute effects of resveratrol. The search terms “resveratrol” were used in combination with “metabolic syndrome”, “obesity”, “diabetes”, “insulin sensitivity”, “plasma glucose” or “visceral fat” in a literature search including papers published up to September 2010. (DOCX) [file pone.0019881.s003.docx]

What is new for an old Molecule? Systematic Review and Recommendations on the use of Resveratrol

Ole Vang, Nihal Ahmad, Clifton A. Baile, Joseph A. Baur, Karen Brown et al.

Supporting information:

| **Table S3:** Effect of resveratrol on obesity and models for diabetes in experimental animals | | | | | | |
| --- | --- | --- | --- | --- | --- | --- |
|  | | | | | | |
| **Species / Strain** | **Treatment** | **Resveratrol dose** | **Duration** | **Effect** | | **References** |
| Insulin sensitivity | | | | | | |
| Male Sprague-Dawley rats | High cholesterol–fructose (HCF) diet for 15  weeks | 1 mg Resv/ kg bw/ day, po | 15 days / 15 weeks | Metabolic characteristics of rats on an HCF diet shifted toward a standard diet.  Insulin-stimulated whole-body glucose uptake ↑  Steady-state glucose uptake of soleus muscle and liver in HCF-fed rats ↑ | | [1] |
| Male Wistar rats | High-fat diet (59% from fat) for 6 weeks | 100 mg Resv/kg bw/ day po | 10 weeks (16 weeks in total) | lipid accumulation in the liver ↓  Abdominal obesity ↓  Insulin resistance ↓  Fasting serum insulin ↓ | | [2] |
| Male Wistar rats | Single ip injection of 50 mg STZ/ kg | 5 mg Resv/ kg bw/ day po | 30 days | Serum insulin↓ | | [3] |
| Lean / Obese Zucker rats |  | 10 mg Resv/ kg /day po | 4 weeks/ 8 weeks | fasting plasma insulin concentration ↓ ( 4 weeks) | | [4] |
| Male C57Bl/6J mice |  | 200 or 400 mg Resv/ kg bw/ day | 9 weeks | Insulin sensitivity ↑ | | [5] |
| male C57BL/6NIA mice | high-calorie diet | 22.4 mg Resv/ kg bw/ day | 6 months | Fasting serum insulin ↓ | | [6] |
| IRS2^-/-^ mice |  | 25mg Resv/ml in drinking water (~ 2.5 mg Resv/ kg bw/ day) | 8 weeks | systemic insulin sensitivity ↑  glucose tolerance → | | [7] |
| AMPKα1^-/-^ and wild-type C57BL/6J mice | Fed a high-fat diet (40% from fat) | 400 mg Resv/ kg bw/ day | 12 weeks | Metabolic rate ↑  Insulin sensitivity → | | [8] |
| C57BL/6 male mice | High-calorie diet (58% from fat) - 14 weeks | 79.2 ng Resv/ day, infused intra cerebro ventricularly. | 5 weeks | Serum insulin ↓ | | [9] |
| Male New Zealand rabbits | Diabetes induced with alloxan (100 mg/kg) and  maintained for 8 weeks | 5 or 50 mg Resv/ L drinking water  (~1.5 or 17 mg/ kg/ day) | 10 weeks, starting 14 days prior alloxan | Serum insulin ↓ | | [10] |
| Blood glucose levels | | | | | | |
| Male Wistar rats | High-fat diet for 30 days | 1 mg Resv/ kg bw/ day via drinking water | 15 days | Serum glucose ↓ | | [11] |
| Male Wistar rats | Single ip injection of 50 mg STZ/ kg | 5 mg Resv/ kg po | 30 days | Serum glucose ↓ | | [3] |
| Sprague–Dawley rats | A single iv injection of 65 mg STZ/kg for 2 weeks | 0.1 or 1 mg Resv/ kg bw/ day +/- 1 mg insulin/ kg bw/ day | 5 days | Plasma glucose ↓ | | [12] |
| Sprague Dawley rats | 65 mg STZ/ kg – 15 days | 2.5 mg Resv/ kg bw/ day | 15 days | Blood glucose ↓ | | [13] |
| Lean / Obese Zucker rats |  | 10 mg Resv/ kg bw/ day po | 4 weeks/ 8 weeks | Fasting levels of glucose ↓ | | [4] |
| Genetically obese mice (Lep^ob/ob^) |  | SRT501 (1,000 mg/ kg) | 3 weeks | Fasting blood glucose ↓ | | [14] |
| Diet-induced obesity (DIO) mice |  | SRT501 (500 mg/ kg) | 4 weeks | Fasting blood glucose ↓  Hyperinsulinaemia in DIO mice ↓ | | [14] |
| Male C57BL/6NIA mice | High-calorie diet | 22.4 mg/ kg / day | 6 months | Fasting glucose ↓ | | [6] |
| Male C57BL/6 mice | High-calorie diet (58% kcal from fat) - 14 weeks | 79.2 ng / day, infused intra cerebro ventricularly | 5 weeks | Blood glucose ↓ | | [9] |
| C57BL/6 mice | Five consecutive ip injections of 55 mg STZ/ kg bw | 20 mg Resv/ kg bw/ day, po | 1 month | Blood glucose ↓ | | [15] |
| Male New Zealand rabbits | Diabetes induced with alloxan (100 mg/ kg bw) and maintained for 8 weeks | 5 or 50 mg Resv/ L drinking water  (~1.5 or 17 mg/ kg bw/ day) | 10 weeks, starting 14 days prior alloxan | Blood glucose → | | [10] |
| Diet-induced obesity | | | | | | |
| Male C57BL/6NIA mice | High-calorie diet | 22.4 mg Resv/ kg bw/ day | 6 months | | Weight gain→ | [6] |
| Male Sprague-Dawley rats | High-caloric diet | 6, 30 or 60 mg Resv/ kg bw/ day | 6 weeks | | Food intake →  Final body weight→  The size of white adipose tissue ↓ | [16] |
| Female Sprague-Dawley rats | High-fat diet (42% from fat) | 20 mg Resv/ kg bw/ day | 8 weeks | | Weight gain →  Food intake of standard diet →  Food intake of high-fat diet ↓ | [17] |
| Lean / Obese Zucker rats |  | 10 mg Resv/ kg bw/ day po | 8 weeks | | Food intake →  Body weight → | [4] |
| Male C57Bl/6J mice |  | 200 or 400 mg/kg bw/day | 9 weeks | | Weight gain ↓  Food intake → | [5] |
| C57BL/6 male mice | high-calorie diet (58% kcal from fat) - 14 weeks | 79.2 ng / day, infused intra cerebro ventricularly | 5 weeks | | Body weight →  Food intake → | [9] |
| AMPKα1^-/-^ and wild-type C57BL/6J mice | Fed a high-fat diet (40% from fat) | 400 mg Resv/ kg bw/ day | 12 weeks | | Food intake →  Body weight of wild-type ↓  AMPKα2^-/-^ mice ↓  AMPKα1^-/-^ mice →  The fat index ↓ in all strains | [8] |
| Grey mouse lemurs |  | 200 mg Resv/ kg bw/ day | 4 weeks | | Weight gain ↓  Food intake ↓ | [18] |
| Visceral fat index and liver mass index | | | | | | |
| Male Wistar rats | high-fat (59% from Fat) – 6 weeks | 100 mg·Resv/ kg bw/ day | 10 week | | Reduced lipid accumulation in liver  Reduced abdominal obesity | [2] |
| Lean / Obese Zucker rats |  | 10 mg Resv/ kg bw/ day, po | 8 weeks | | In obese rats, abdominal fat ↓  plasma triglycerides ↓  Free fatty acids ↓  Total cholesterol ↓ | [4] |
| Male Wistar CRL: Wi (Han) | High carbohydrate - fat free modified diet + induction of steatosis | 10 mg Resv daily po (~ 44 mg/ kg bw/ day) | 4 weeks | | Grade of steatosis ↓ | [19] |
| DIO: Diet-induced obesity; HCF: high cholesterol–fructose; STZ: streptozotocin  bw: body weight; iv: intravenous; ip: intraperitoneally; po: per oral  Effect are indicated by ↓: reduction; ↑: enhancement; →: no effect. | | | | | | |

**References**

1. Deng JY, Hsieh PS, Huang JP, Lu LS, Hung LM (2008) Activation of estrogen receptor is crucial for resveratrol-stimulating muscular glucose uptake via both insulin-dependent and -independent pathways. Diabetes 57: 1814-1823.

2. Shang J, Chen LL, Xiao FX, Sun H, Ding HC et al. (2008) Resveratrol improves non-alcoholic fatty liver disease by activating AMPactivated protein kinase. Acta Pharmacol Sin 29: 698-706.

3. Palsamy P, Subramanian S (2008) Resveratrol, a natural phytoalexin, normalizes hyperglycemia in streptozotocin-nicotinamide induced experimental diabetic rats. Biomed Pharmacother 62: 598-605.

4. Rivera L, Moron R, Zarzuelo A, Galisteo M (2009) Long-term resveratrol administration reduces metabolic disturbances and lowers blood pressure in obese Zucker rats. Biochem Pharmacol 77: 1053-1063.

5. Lagouge M, Argmann C, Gerhart-Hines Z, Meziane H, Lerin C et al. (2006) Resveratrol improves mitochondrial function and protects against metabolic disease by activating SIRT1 and PGC-1alpha. Cell 127: 1109-1122.

6. Baur JA, Pearson KJ, Price NL, Jamieson HA, Lerin C et al. (2006) Resveratrol improves health and survival of mice on a high-calorie diet. Nature 444: 337-342.

7. Gonzalez-Rodriguez A, Mas Gutierrez JA, Sanz-Gonzalez S, Ros M, Burks DJ et al. (2010) Inhibition of PTP1B restores IRS1-mediated hepatic insulin signaling in IRS2-deficient mice. Diabetes 59: 588-599.

8. Um JH, Park SJ, Kang H, Yang S, Foretz M et al. (2010) AMP-activated protein kinase-deficient mice are resistant to the metabolic effects of resveratrol. Diabetes 59: 554-563.

9. Ramadori G, Gautron L, Fujikawa T, Vianna CR, Elmquist JK et al. (2009) Central administration of resveratrol improves diet-induced diabetes. Endocrinology 150: 5326-5333.

10. Akar F, Pektas MB, Tufan C, Soylemez S, Sepici A et al. (2011) Resveratrol shows vasoprotective effect reducing oxidative stress without affecting metabolic disturbances in insulin-dependent diabetes of rabbits. Cardiovasc Drugs Ther .

11. Rocha KK, Souza GA, Ebaid GX, Seiva FR, Cataneo AC et al. (2009) Resveratrol toxicity: effects on risk factors for atherosclerosis and hepatic oxidative stress in standard and high-fat diets. Food Chem Toxicol 47: 1362-1367.

12. Huang JP, Huang SS, Deng JY, Chang CC, Day YJ et al. (2010) Insulin and resveratrol act synergistically, preventing cardiac dysfunction in diabetes, but the advantage of resveratrol in diabetics with acute heart attack is antagonized by insulin. Free Radic Biol Med 49: 1710-1721.

13. Thirunavukkarasu M, Penumathsa SV, Koneru S, Juhasz B, Zhan L et al. (2007) Resveratrol alleviates cardiac dysfunction in streptozotocin-induced diabetes: Role of nitric oxide, thioredoxin, and heme oxygenase. Free Radic Biol Med 43: 720-729.

14. Milne JC, Lambert PD, Schenk S, Carney DP, Smith JJ et al. (2007) Small molecule activators of SIRT1 as therapeutics for the treatment of type 2 diabetes. Nature 450: 712-716.

15. Kim YH, Kim YS, Kang SS, Cho GJ, Choi WS (2010) Resveratrol inhibits neuronal apoptosis and elevated Ca2+/calmodulin-dependent protein kinase II activity in diabetic mouse retina. Diabetes 59: 1825-1835.

16. Macarulla MT, Alberdi G, Gomez S, Tueros I, Bald C et al. (2009) Effects of different doses of resveratrol on body fat and serum parameters in rats fed a hypercaloric diet. J Physiol Biochem 65: 369-376.

17. Aubin MC, Lajoie C, Clement R, Gosselin H, Calderone A et al. (2008) Female rats fed a high-fat diet were associated with vascular dysfunction and cardiac fibrosis in the absence of overt obesity and hyperlipidemia: therapeutic potential of resveratrol. J Pharmacol Exp Ther 325: 961-968.

18. Dal-Pan A, Blanc S, Aujard F (2010) Resveratrol suppresses body mass gain in a seasonal non-human primate model of obesity. BMC Physiol 10: 11.

19. Bujanda L, Hijona E, Larzabal M, Beraza M, Aldazabal P et al. (2008) Resveratrol inhibits nonalcoholic fatty liver disease in rats. BMC Gastroenterol 8: 40.
