# Supplementary material for: What Is New for an Old Molecule? Systematic Review and Recommendations on the Use of Resveratrol
Source: PLoS One. 2011 Jun 16;6(6):e19881. doi: 10.1371/journal.pone.0019881 (PMC3116821; doi:10.1371/journal.pone.0019881)
Supplement: Table S4 — Overview of the neuroprotective effect by resveratrol was in focus of 163 papers, of which 40 papers concerned animal with acute exposure and sub- and chronic exposure. The search terms “resveratrol” was used in combination with “neuroprotection” or “neuroprotective” including papers published up to September 2010. (DOCX) [file pone.0019881.s004.docx]

What is new for an old Molecule? Systematic Review and Recommendations on the use of Resveratrol

Ole Vang, Nihal Ahmad, Clifton A. Baile, Joseph A. Baur, Karen Brown et al.

Supporting information:

| **Table S4:** Neuroprotective effect of resveratrol (acute, sub chronic and chronic exposure) | | | | | |
| --- | --- | --- | --- | --- | --- |
|  | | | | | |
| **Animal** | **Treatment** | **Resveratrol dose** | **Duration** | **Effect** | **References** |
| Acute models | | | | | |
| Wistar male rats | Weight-drop method was used for achieving head trauma | 100 mg/ kg bw, single dose of Resv | Acute exposure | Tissue lesion area ↓ | [1] |
| Wistar male rats | Weight-drop trauma modeling | 100 mg Resv/ kg bw,  ip immediately after trauma | Acute exposure | Recovery of impaired motor function score ↑  Recovery of impaired inclined plane score ↑  Spinal cord injury area ↓ | [2] |
| Long-Evans rats | Focal cerebral ischemia induced by occlusion of the middle cerebral artery (MCA) for 1 hour | 0.1 and 1 µg Resv/ kg bw was iv injected after 1 hour MCA occlusion | Acute exposure | Infarct volume ↓ by 1 µg/kg Resv | [3] |
| Sprague–Dawley male rats | Right middle cerebral  artery occlusion (MCAO) | Systemic inject. of Resv ( 2x10^-3^, 2x10^-4^, 1x10^-4^  mg/ kg bw) | Acute exposure | Infarct area ↓  MCAO induced renal sympathetic nerve activity ↓ | [4] |
| Wistar male rats | Middle cerebral artery occlusion (MCAO) | 100 mg Resv/kg bw administrated iv, 15 min before occlusion and the time of reperfusion | Acute exposure | MCAO-induced brain edema↓  MCAO-induced cerebral ischemia ↓ | [5] |
| Wistar male rats | Cerebral ischemia induced by bilateral cerebral artery ligation | iv: 20 mg Resv/ kg bw | Single exposure | Ischemia induced neuronal damage scorea in hippocampus ↓  Ischemia induced SOD ↓  NO ↑ | [6] |
| Sprague–Dawley rats | Asphyxial cardiac arrest (ACA) | ip: 50 or 100 mg Resv/ kg bw 48 h before the induction of ACA | Acute exposure | Tolerance against brain injury ↑ | [7] |
| Male Wistar rats | Kainate-induced epileptic rats (intra-hippocampal injections) | ig: 15 mg Resv/ kg | Acute exposure | Frequency of spontaneous seizures ↓  Epileptiform discharges↓  counteract kainate-induced neuronal cell death | [8] |
| Sprague-Dawley rats | Spinal cord injured | 50 and 100 mg Resv/ kg bw (ip) immediately after trauma | Acute exposure | Edema-induced cell death ↓  Edema-induced lipid peroxidation ↓ | [9] |
| Wistar rats (7-day-old pups) | Percussion trauma  model in immature rats | 100 mg/kg resveratrol (ip) immediately after trauma | Acute exposure | Trauma-induced decreased locomotor activity ↑  Trauma-induced decreased memory discrimination index ↑ | [10] |
| Male Swiss mice | Induction of neurotoxicity by 40 mg/ kg bw dose of lindane | 5 mg Resv/kg in combination with other antioxidants  50 mg/kg Vitamin C  20 mg/kg α-lipoic acid  50 mg/kg Vitamin E | Acute exposure | Lindane-decreased level of AChE in cerebellum and pons-medulla  Antioxidants: ↑  Lindane-decreased level of butyryl cholinesterase in cerebellum and pons-medulla  Antioxidants:↑ | [11] |
| New Zealand rabbits | Spinal cord ischemia - occlusion of the infrarenal aorta | 1 or 10 Resv mg/kg given 30 min before operation | Acute exposure | Paraplegia was significantly counteracted by 20 mg Resv/ kg | [12] |
| Sub chronic / chronic exposure | | | | | |
| Wistar male rats | Middle cerebral artery occlusion (MCAO) | 20 mg Resv/ kg bw daily (ip) before MCAO | 21 days | MCAO reduced grip score ↑  MCAO reduced time on rota rod ↑  MCAO reduced locomotor activity ↑  MCAO induced lipid peroxidation ↓  MCAO induced GSH ↓  MCAO infarct volume ↓ | [13] |
| Sprague-Dawley male rats | Controlled cortical impact (CCI) model | 100 mg Resv /kg bw (ip) | 3 days | Motor performance ↑  Visuospatial memory ↑ | [14] |
| Sprague–Dawley male rats | Middle cerebral artery occlusion (MCAO) | 30 mg Resv /kg bw (ip) | 7 days | MCAO induced infarct ↓  MCAO induced neurological deficit ↓ | [15] |
| Sprague Dawley male rats | Kainic acid (8 mg/kg bw) daily for 5 days caused neuronal death and activation of astrocytes and microglial cells | 30 mg Resv/kg bw/  day | 5 days | Neuronal death ↓  Activation of astrocytes ↓ Activation of microglial cells ↓ | [16] |
| Sprague–Dawley rats | Injection of 6-hydroxy-dopamine (6-OHDA) into the right striatum | 10, 20 or 40 mg Resv/ kg bw/ day (po) | 10 weeks | 6-OHDA-induced contra lateral turns ↓ | [17] |
| Wistar male rats | Injection of 10 μg 6-OHDA (ig) | Daily injection with 20 mg Resv/ kg bw (ip) | 15 days | 6-OHDA-induced contralateral rotations ↓  6-OHDA-depleted muscles coordination ↑  6-OHDA-impairment in the adjusting steps ↑ | [18] |
| Sprague–Dawley male rats | Daily i.p. injection with 10 mg/kg 3-nitropropionic acid (3-NPA) | Daily ip injection with 100 mg Resv/ kg | 4 weeks | 3-NPA-induced paresis ↓  3-NPA-reduced motor nerve conductivity ↑ | [19] |
| Wistar male rats | ip administration of NPA (20 mg/kg bw for 4 days) | 5 or 10 mg Resv/ kg bw, po, from day 4 before injection of NPA | 8 days | 3NPA- induced motor impairment ↓  3NPA- induced cognitive impairment ↓ | [20] |
| Sprague–Dawley male rats | 55 mg STZ/ kg bw  (ip) | Daily 10 or 20 mg Resv/ kg bw | 2 weeks | Diabetes induced motor conduction velocity (MCV) ↑ | [21] |
| Sprague–Dawley male rats | 55 mg STZ/ kg bw  (ip) | Daily 10 mg Resv/ kg bw | 2 weeks | Diabetes reduced MCV ↑  Diabetes reduced nerve blood flow ↑ | [22] |
| Wistar male rats | 60 mg STZ/ kg bw  (ip) | Daily 10 mg Resv/ kg bw (ip) | 6 weeks | In regions of the central nervous system:  STZ-reduced GSH ↑  STZ-induced lipid peroxidation ↓ | [23] |
| Wistar male rats | 55 mg STZ/ kg bw (ip) | Daily 10 or 20 mg Resv/ kg bw (po) | 30 days | STZ-induced AChE activity in cerebral cortex synaptosomes ↓ | [24] |
| Wistar male rats | Intracerebroventricular administration of colchicine (15 μg) | 10 and 20 mg Resv/ kg bw (po) beginning 4 days prior to colchicine injection | 25 days | Colchicine-induced cognitive impairment ↑ | [25] |
| Wistar rats | Induction of vasospasm: autologous blood (0.3 mL) was injected into the cisterna magna. | iv injection of 10 mg Resv/ kg bw/ day | 3 days | Relaxation of smooth muscle in the wall of the basilar artery ↑  Neuroprotection against cerebral ischemia ↑ | [26] |
| Wistar male rats |  | Daily 1.25 – 25 mg Resv/ kg bw (ip) | 7 days | Lipid peroxidation in brain ↓  Antioxidative enzymes ↑ | [27] |
| p25-CK transgenic mice on a C57BL6 background |  | Intra cerebro ventricular injection (2.5 µg Resv) every 2-3 day | 3 weeks | Associative learning was  rescued  Neurons survival ↑ | [28] |
| Male Balb/c mice | Injury induced by MCA occlusion and reperfusion | 50 mg/kg bw/ day, gavages | 7 days | Mean neurologic scores ↓  infarct volumes of the ischemia and reperfusion groups↓ | [29] |
| C57BL/6 mice | Injury induced by MCA occlusion and reperfusion | 20 mg Resv / kg bw/ day | 7 days | MCA-induced infarct volumes ↓ | [30] |
| C57BL/6 mice | 1-methyl-4-phenyl-  1,2,3,6-tetrahydro-pyridine (MPTP) targets nigrostriatal dopaminergic neurons | 50 or 100 mg Resv/ kg bw/ day | 1 or 2 weeks | MPTP-induced depletion of striatal dopamine ↑  Rescue nigral neurons from MPTP insults ↑ | [31] |
| Male Balb/C mice | MPTP treatment (30 mg/kg, ip) | 20 mg Resv/ kg bw/ day, iv | 7 days | Motor coordination ↑  MPTP-induced muscle rigidity ↑ | [32] |
| Male C57BL/6 | MPTP treatment (4 injections of 7 or 10 mg/kg, ip) | 50 mg Resv/ kg/ day, gavage  100 mg Resv/ kg/ day, gavage | 7 days /  14 days before MPTP | MPTP-reduced striatal dopamine ↑  MPTP-reduced striatal tyrosine hydroxylase ↑ | [31] |
| SJL/J mice | Induction of EAE by proteolipid protein peptide | Intravitreal injections of 5, 10 and 80 pmol SRT501 on day 0, 3, 7 and 11. | 2 weeks | Acute loss of retinal ganglion cell ↑  Optic nerve inflammation → | [33] |
| Male C57BL/6 mice | Diabetes induced by 55 mg STZ/ kg bw (ip), once a day for 5 days | 20 mg Resv/ kg/ day by gavages | 4 weeks | STZ induced retinal cell death ↓ | [34] |
| C57BL/6 mice | Standard diet or high fat diet | ~ 200 mg Resv/ kg/ day in diet or by mini pump | 4 weeks | Brain MnSOD level and activity in high fat mice ↑  Brain catalase activity →  Brain GSH Peroxidase activity in high fat mice ↓ | [35] |
| Offspring of Tg19959 crossed with C57/B6SJL |  | 0.2% Resv in diet (~300 mg/ kg bw/ day) | 45 days | Plaque formation ↓ | [36] |
| ddY Mice | Permanent MCAO | 20 mg Resv/ kg bw/ day | 3 days | Brain infarct volume ↓ | [37] |
| C57BL/6N female mice | Middle age (12–15-months-old) exposed to 0.76 g EtOH /kg bw) | 1.15 mg Resv/ day in the drinking water (~44 mg/ kg bw + 0.71 g EtOH /kg bw | 6 weeks | Cued learning: : EtOH+Resv relative to EtOH →  Spatial learning: EtOH+Resv relative to EtOH ↑ | [38] |
| male C57Bl/6 mice | 24 month age mice | 0.15 mg Resv/ g diet (~ 18 mg/ kg bw), starting when 12 month old | 12 month | Acquisition of a spatial Y-maze test ↑ | [39] |
| ACA: Asphyxial cardiac arrest; AChE: Acetylcholine esterase; EAE: Experimental autoimmune encephalomyelitis; GSH: Reduced glutathione; MCAO: middle cerebral artery occlusion; MCV: motor conduction velocity; 3NPA: 3-nitropropionic acid; MnSOD: Mn-superoxide dismutase; MPTP: 1-methyl-4-phenyl-1,2,3,6-tetrahydro-pyridine; 6-OHDA: 6-hydroxydopamine; STZ: streptozotocin;  ig: intra gastrically; iv: intravenous; po: per oral;  Effect are indicated by ↓: reduction; ↑: enhancement; →: no effect. | | | | | |

**References**

1. Ates O, Cayli S, Altinoz E, Gurses I, Yucel N et al. (2007) Neuroprotection by resveratrol against traumatic brain injury in rats. Mol Cell Biochem 294: 137-144.

2. Ates O, Cayli S, Altinoz E, Gurses I, Yucel N et al. (2006) Effects of resveratrol and methylprednisolone on biochemical, neurobehavioral and histopathological recovery after experimental spinal cord injury. Acta Pharmacol Sin 27: 1317-1325.

3. Tsai SK, Hung LM, Fu YT, Cheng H, Nien MW et al. (2007) Resveratrol neuroprotective effects during focal cerebral ischemia injury via nitric oxide mechanism in rats. J Vasc Surg 46: 346-353.

4. Saleh MC, Connell BJ, Saleh TM (2010) Resveratrol preconditioning induces cellular stress proteins and is mediated via NMDA and estrogen receptors. Neuroscience 166: 445-454.

5. Yousuf S, Atif F, Ahmad M, Hoda N, Ishrat T et al. (2009) Resveratrol exerts its neuroprotective effect by modulating mitochondrial dysfunctions and associated cell death during cerebral ischemia. Brain Res 1250: 242-253.

6. Lu KT, Chiou RY, Chen LG, Chen MH, Tseng WT et al. (2006) Neuroprotective effects of resveratrol on cerebral ischemia-induced neuron loss mediated by free radical scavenging and cerebral blood flow elevation. J Agric Food Chem 54: 3126-3131.

7. Della-Morte D, Dave KR, Defazio RA, Bao YC, Raval AP et al. (2009) Resveratrol pretreatment protects rat brain from cerebral ischemic damage via a sirtuin 1 -- uncoupling protein 2 pathway. Neuroscience 159: 993-1002.

8. Wu Z, Xu Q, Zhang L, Kong D, Ma R et al. (2009) Protective effect of resveratrol against kainate-induced temporal lobe epilepsy in rats. Neurochem Res 34: 1393-1400.

9. Yang YB, Piao YJ (2003) Effects of resveratrol on secondary damages after acute spinal cord injury in rats. Acta Pharmacol Sin 24: 703-710.

10. Sonmez U, Sonmez A, Erbil G, Tekmen I, Baykara B (2007) Neuroprotective effects of resveratrol against traumatic brain injury in immature rats. Neurosci Lett 420: 133-137.

11. Bist R, Bhatt DK (2010) Augmentation of cholinesterases and ATPase activities in the cerebellum and pons-medulla oblongata, by a combination of antioxidants (resveratrol, ascorbic acid, alpha-lipoic acid and vitamin E), in acutely lindane intoxicated mice. J Neurol Sci 296: 83-87.

12. Kiziltepe U, Turan NN, Han U, Ulus AT, Akar F (2004) Resveratrol, a red wine polyphenol, protects spinal cord from ischemia-reperfusion injury. J Vasc Surg 40: 138-145.

13. Sinha K, Chaudhary G, Gupta YK (2002) Protective effect of resveratrol against oxidative stress in middle cerebral artery occlusion model of stroke in rats. Life Sci 71: 655-665.

14. Singleton RH, Yan HQ, Fellows-Mayle W, Dixon CE (2010) Resveratrol attenuates behavioral impairments and reduces cortical and hippocampal loss in a rat controlled cortical impact model of traumatic brain injury. J Neurotrauma 27: 1091-1099.

15. Li C, Yan Z, Yang J, Chen H, Li H et al. (2010) Neuroprotective effects of resveratrol on ischemic injury mediated by modulating the release of neurotransmitter and neuromodulator in rats. Neurochem Int 56: 495-500.

16. Wang Q, Yu S, Simonyi A, Rottinghaus G, Sun GY et al. (2004) Resveratrol protects against neurotoxicity induced by kainic acid. Neurochem Res 29: 2105-2112.

17. Jin F, Wu Q, Lu YF, Gong QH, Shi JS (2008) Neuroprotective effect of resveratrol on 6-OHDA-induced Parkinson's disease in rats. Eur J Pharmacol 600: 78-82.

18. Khan MM, Ahmad A, Ishrat T, Khan MB, Hoda MN et al. (2010) Resveratrol attenuates 6-hydroxydopamine-induced oxidative damage and dopamine depletion in rat model of Parkinson's disease. Brain Res 1328: 139-151.

19. Binienda ZK, Beaudoin MA, Gough B, Ali SF, Virmani A (2010) Assessment of 3-nitropropionic acid-evoked peripheral neuropathy in rats: neuroprotective effects of acetyl-L-carnitine and resveratrol. Neurosci Lett 480: 117-121.

20. Kumar P, Padi SS, Naidu PS, Kumar A (2006) Effect of resveratrol on 3-nitropropionic acid-induced biochemical and behavioural changes: possible neuroprotective mechanisms. Behav Pharmacol 17: 485-492.

21. Kumar A, Sharma SS (2010) NF-kappaB inhibitory action of resveratrol: A probable mechanism of neuroprotection in experimental diabetic neuropathy. Biochem Biophys Res Commun 394: 360-365.

22. Sharma SS, Kumar A, Arora M, Kaundal RK (2009) Neuroprotective potential of combination of resveratrol and 4-amino-1,8-naphthalimide in experimental diabetic neuropathy: focus on functional, sensorimotor and biochemical changes. Free Radic Res 43: 400-408.

23. Ates O, Cayli SR, Yucel N, Altinoz E, Kocak A et al. (2007) Central nervous system protection by resveratrol in streptozotocin-induced diabetic rats. J Clin Neurosci 14: 256-260.

24. Schmatz R, Mazzanti CM, Spanevello R, Stefanello N, Gutierres J et al. (2009) Ectonucleotidase and acetylcholinesterase activities in synaptosomes from the cerebral cortex of streptozotocin-induced diabetic rats and treated with resveratrol. Brain Res Bull 80: 371-376.

25. Kumar A, Naidu PS, Seghal N, Padi SS (2007) Neuroprotective effects of resveratrol against intracerebroventricular colchicine-induced cognitive impairment and oxidative stress in rats. Pharmacology 79: 17-26.

26. Karaoglan A, Akdemir O, Barut S, Kokturk S, Uzun H et al. (2008) The effects of resveratrol on vasospasm after experimental subarachnoidal hemorrhage in rats. Surg Neurol 70: 337-343.

27. Mokni M, Elkahoui S, Limam F, Amri M, Aouani E (2007) Effect of resveratrol on antioxidant enzyme activities in the brain of healthy rat. Neurochem Res 32: 981-987.

28. Kim D, Nguyen MD, Dobbin MM, Fischer A, Sananbenesi F et al. (2007) SIRT1 deacetylase protects against neurodegeneration in models for Alzheimer's disease and amyotrophic lateral sclerosis. EMBO J 26: 3169-3179.

29. Dong W, Li N, Gao D, Zhen H, Zhang X et al. (2008) Resveratrol attenuates ischemic brain damage in the delayed phase after stroke and induces messenger RNA and protein express for angiogenic factors. J Vasc Surg 48: 709-714.

30. Sakata Y, Zhuang H, Kwansa H, Koehler RC, Dore S (2010) Resveratrol protects against experimental stroke: Putative neuroprotective role of heme oxygenase 1. Exp Neurol 224: 325-329.

31. Blanchet J, Longpre F, Bureau G, Morissette M, Dipaolo T et al. (2008) Resveratrol, a red wine polyphenol, protects dopaminergic neurons in MPTP-treated mice. Prog Neuropsychopharmacol Biol Psychiatry 32: 1243-1250.

32. Lu KT, Ko MC, Chen BY, Huang JC, Hsieh CW et al. (2008) Neuroprotective Effects of Resveratrol on MPTP-Induced Neuron Loss Mediated by Free Radical Scavenging. J Agric Food Chem 56: 6910-6913.

33. Shindler KS, Ventura E, Rex TS, Elliott P, Rostami A (2007) SIRT1 activation confers neuroprotection in experimental optic neuritis. Invest Ophthalmol Visual Sci 48: 3602-3609.

34. Kim YH, Kim YS, Kang SS, Cho GJ, Choi WS (2010) Resveratrol inhibits neuronal apoptosis and elevated Ca2+/calmodulin-dependent protein kinase II activity in diabetic mouse retina. Diabetes 59: 1825-1835.

35. Robb EL, Winkelmolen L, Visanji N, Brotchie J, Stuart JA (2008) Dietary resveratrol administration increases MnSOD expression and activity in mouse brain. Biochem Biophys Res Commun 372: 254-259.

36. Karuppagounder SS, Pinto JT, Xu H, Chen LH, Beal MF et al. (2009) Dietary supplementation with resveratrol reduces plaque pathology in a transgenic model of Alzheimer's disease. Neurochem Int 54: 111-118.

37. Inoue H, Jiang XF, Katayama T, Osada S, Umesono K et al. (2003) Brain protection by resveratrol and fenofibrate against stroke requires peroxisome proliferator-activated receptor alpha in mice. Neurosci Lett 352: 203-206.

38. Ranney A, Petro MS (2009) Resveratrol protects spatial learning in middle-aged C57BL/6 mice from effects of ethanol. Behav Pharmacol 20: 330-336.

39. Oomen CA, Farkas E, Roman V, van der Beek EM, Luiten PG et al. (2009) Resveratrol preserves cerebrovascular density and cognitive function in aging mice. Front Aging Neurosci 1:4.: 4.
