# Supplementary material for: What Is New for an Old Molecule? Systematic Review and Recommendations on the Use of Resveratrol
Source: PLoS One. 2011 Jun 16;6(6):e19881. doi: 10.1371/journal.pone.0019881 (PMC3116821; doi:10.1371/journal.pone.0019881)
Supplement: Table S5 — Overview of the effect of resveratrol on inflammatory markers in experimental animals. To identify papers investigating the effect of resveratrol on inflammatory markers, a literature search using the terms “resveratrol” in combination with “inflammation” or “inflammatory” was done, including papers published up to September 2010. In total, 559 papers were identified, whereas only 31 papers included sub-chronic and chronic exposures of resveratrol to experimental animals. (DOCX) [file pone.0019881.s005.docx]

What is new for an old Molecule? Systematic Review and Recommendations on the use of Resveratrol

Ole Vang, Nihal Ahmad, Clifton A. Baile, Joseph A. Baur, Karen Brown et al.

Supporting information:

| **Table S5:** Effect of resveratrol on inflammatory markers | | | | | |
| --- | --- | --- | --- | --- | --- |
|  | | | | | |
| **Animal** | **Inducer** | **Resveratrol dose** | **Duration** | **Effect** | **References** |
| Male Wistar rats | Colonic anastomosis | 0.5 mg Resv/ kg, po | 7 d before operation | Plasma IL-6: 3 d after anastomosis: ↑  0 , 5 or 7 d after: →  Tissue NF-κB: 3 and 5 d: ↓ 0, 7 d → | [1] |
| Male Wistar rats | Chronic colonic injury was induced by intra colonic instillation of TNBS | 10 mg Resv/ kg bw/ day, po, staring 24 h after TNBS treatment | 2 weeks | Microscopic damage: Resv↓  TNFα: TNBS↑, TNBS+Resv↓  NF-κBp65: TNBS↑, TNBS+Resv↓  Cox-2: TNBS↑, TNBS+Resv↓  PGE2: TNBS↑, TNBS+Resv↑↑  PGD2: TNBS↑, TNBS+Resv→ | [2] |
| Male Fischer F344 rats | Colitis induced by 5% DSS | 1 mg Resv/ kg bw/ day | 25 days | DSS-induced inﬁltration of inﬂammatory cells ↓  DSS induced PGE2 level ↓ | [3] |
| Male Wistar rats | 20 mg DMH /kg bw, once a week for 15 weeks | 8 mg Resv/ kg bw/ day, simultaneously with DMH, after DMH treatment or in the entire period | 15 / 30 weeks | Colon COX-2 expression:  DMH↑, DMH+Resv ↓ | [4] |
| Male Sprague-Dawley rats | ip injections of 40 mg DMN/ kg bw causing liver fibrosis | 10 mg Resv/ kg bw/ day | 7 d after DMN treatment | IL-1β: DMN↑, DMN+Resv↓  TNFα: DMN↑, DMN+Resv↓  iNOS: DMN↑, DMN+Resv↓ | [5] |
| Male Sprague–Dawley rats | Diabetes induced by 55 mg STZ/ kg bw, ip | resveratrol 10 and 20 mg/ kg bw/ day, 6 weeks  after diabetes induction | 2 weeks | TNFα: STZ↑, STZ+Resv↓  IL-6: STZ↑, STZ+Resv↓  NF-κBp65: STZ↑, STZ+Resv↓ | [6] |
| Male Wistar rats | Diabetes was induced 50 mg STZ/ kg bw | 5 mg Resv/ kg bw/ day | 30 days | In plasma and liver:  TNFα: STZ↑, STZ+Resv↓  IL-1β: STZ ↑, STZ+Resv↓  IL-6: STZ↑, STZ+Resv↓ | [7] |
| Wistar rats | 20 mg MCT/ kg bw, ip | 10 mg Resv/ kg bw/day, po | 5 days | Plasma TNFα: MCT↑, MCT+Resv↓ | [8] |
| Sprague-Dawley rats | MCT induced hypertension | 25 mg Resv/ kg bw/ day, po, from day 1 post MCT |  | mRNA expression of  IL-6: MCT↑, MCT+Resv↓  IL-1: MCT↑, MCT+Resv↓  TNFα: MCT↑, MCT+Resv↓  PDGFα: MCT↑, MCT+Resv↓  PDGFβ: MCT↑, MCT+Resv↓  TGFβ: MCT↑, MCT+Resv→  MCP-1: MCT↑,MCT+Resv↓ | [9] |
| Lean / Obese Zucker rats |  | 10 mg Resv/ kg bw/ day, po | 8 weeks | TNFα: OBS↑, OBS+Resv↓ | [10] |
| Male Wistar rats | Exposed to cigarette smoke (CS) for 1 week | 25 mg Resv/ kg bw/ day in drinking water | 2 days pre-treatment | IL-1: CS↑, CS+Resv↓  IL-6: CS↑, CS+Resv↓  ICAM1: CS↑, CS+Resv↓  iNOS: CS↑, CS+Resv↓  TNFα: CS↑, CS+Resv→ | [11] |
| Sprague–Dawley rats | 6-OHDA injected into the right striatum | 10, 20 and 40 mg Resv/ kg was given orally | 10 weeks | Cox-2 expression in the substantia: nigra: 6-OHDA↑, 6-OHDA+Resv↓ | [12] |
| Male Wistar CRL: Wi (Han) rats | Steatosis (ST) by a high carbohydrate-fat free modified diet 4 days per week, and fasted for the remaining 3 days (4 weeks) | 10 mg Resv, po (~ 44 mg/ kg bw/ day) | 4 weeks | TNFα: ST↑, ST+Resv↓ | [13] |
| Male Wistar rats | Idiopathic pulmonary ﬁbrosis induced a single dose of 5mg BLE/ kg | 10 mg Resv/ kg bw/ day | 2 weeks | TNFα: BLE↑, BLE+Resv↓  IL-1β: BLE ↑, BLE +Resv↓  IL-6: BLE ↑, BLE+Resv↓  TGFβ: BLE ↑, BLE+Resv↓ | [14] |
| Male Swiss rats | Bile duct ligation (BDL) | 10 mg Resv/ kg, ip, once a day | 28 days | IL-1β: BDL↑, BDL+Resv↓  IL-6: BDL↑, BDL+Resv↓  TNFα: BDL↑, BDL+Resv↓ | [15] |
| Male and female C57BL/6 mice | Water containing 1% DSS | Resv in diet at 75 to 300 ppm (~ 12 to 48 mg/ kg bw/ day) | 62 days | T cells expressing  TNFα: DSS↑, DSS+Resv↓  IFNγ: DSS↑, DSS+Resv↓ | [16] |
| Female Swiss mice | Ehrlich ascites carcinoma cells from a spontaneous mammary cancer (EAC) | 20 or 40 mg Resv/ kg bw/ day, ip | 20 days | CRP: EAC↑, EAC+Resv↓ (20 & 40 mg)  TNFα: EAC ↑, EAC +Resv↓ (20 & 40 mg) | [17] |
| C57BL/6J mice | Colitis was induced for the last 8 days by 1% DSS in drinking water | 2.1 mg Resv/ kg bw/ day | 29 days | TNFα: DSS→, DSS+Resv→  IL-1β: DSS→, DSS+Resv→  IL-6: DSS ↑, DSS +Resv↓  IL-10: DSS ↑, DSS+Resv↓  IL-3: DSS ↑, DSS+Resv↑  sTNF RI, p55 subunit: DSS ↑, DSS +Resv↓ | [18] |
| Female C57BL/6 mice | 3% DSS in the drinking water for 5 days | Resveratrol in diet at 20 mg/kg (~ 3 mg/ kg bw/ day) | 26 days | TNFα: DSS↑, DSS+Resv↓  IL-1β: DSS↑, DSS+Resv↓  IL-10: DSS↓, DSS+Resv↑  PGES-1: DSS↑, DSS+Resv↓  COX2: DSS↑, DSS+Resv↓  iNOS: DSS↑, DSS+Resv↓ | [19] |
| Female C57BL/6 mice | Drinking water containing 3% DSS | 10, 50, or 100 mg Resv/ kg bw/ day, po | 14 days | TNFα: DSS↑, DSS+ Resv ↓  IL-1β: DSS↑, DSS+ Resv ↓  IL-6: DSS↓, DSS+ Resv ↑  IFNγ: DSS↑, DSS+ Resv ↓  COX2: DSS↑, DSS+ Resv ↓  iNOS: DSS↑, DSS+ Resv ↓ | [20] |
| Male BALB/c mice | Ulcerative colitis induced by 5% DSS in drinking water for 7 days | 30 or 60 mg Resv/ kg bw/ day | 14 days | TNF-α: DSS↑, DSS+ Resv ↓  IL-8: DSS↑, DSS+ Resv ↓  IFN-γ: DSS↑, DSS+ Resv ↓  p22phox: DSS↑, DSS+Resv↓  gp91phox DSS↑, DSS+Resv↓ | [21] |
| Control: heterozygote m-  Lepr^db^ mice | type 2 diabetes: homozygous Lepr^db^ mice | 20 mg Resv/ kg bw/ day po | 4 weeks | TNF-α: Lepr^db-/-^↑, Lepr^db-/-^+Resv ↓ | [22] |
| C57/B6 mice | Angiotensin II (Ang II) | Resv in drinking water at 0.1 mg/ml (~10 mg Resv/ kg bw/ day | 4 weeks | IL-6: Ang II↑, Ang II+Resv↓ | [23] |
| Female BALB/c mice | Asthma sensitization plus challenge with ovalbumin (OVA) | 30 mg Resv/ kg bw, po | 32 days | Recruitment of leukocytes in lung tissue: OVA↑, OVA+Resv↓  IL-4: OVA↑, OVA+Resv↓  IL-5: OVA↑, OVA+Resv↓ | [24] |
| BALB-c mice of either sex | 100 mg Naphthalene (NAP)/ kg bw/ day, ip for 30 days | 10 mg Resv/ kg bw/ day, po | 30 days | TNFα: NAP↑, NAP+Resv↓  IL-1β: NAP↑, NAP+Resv↓  IL-6: NAP↓, NAP+Resv↑ | [25] |
| BALB/c mice | Injected L1210 cells, ip | 12.5, 25, 50 mg Resv/ kg /day, ig | 3 weeks | IL-6: L1210↑, L1210+Resv↓ | [26] |
| Apolipoprotein E KO mice |  | P183/1-mixture: 27% Resv, 1.37 % caffeic acid and 8.35% cathechin | 8 weeks | In vascular wall:  MCP-1↓; MIPα↓, MIPβ↓, IL6↓; IL10→ | [27] |
| Male Laka mice | Diabetes induced by 200 mg STZ/ kg | 20 mg Resv/ kg bw/ day,  po | 4 weeks | TNFα: STZ↑, STZ+Resv↓, Insulin+ STZ+Resv↓↓ | [28] |
| C57BL/6 mice | EAE: sc immunization with 100 µl of 20 or 150 mg myelin oligoden-drocyte glycoprotein | 100 mg Resv/ kg bw/ day, po | 32 days | EAE+Resv relative to EAE: IL12p40↓, IL13↓, IL17↑, G-CSF↑, MIP1a↓, MCP-1↓, RANTES↓ | [29] |
| Female SKH-1 hairless mice | UVB irradiation | 25 mol Resv in 200 µl ace-  tone per mouse |  | ODC: UVB↑, UVB+Resv↓  COX: UVB↑, UVB+Resv↓ | [30] |
| Balb/C mice | Injected mouse hepatocellular carcinoma  cells H22  On day 11, 0.1 mg LPS/ kg, ip; the mice were killed 90 min later | ip injection with Resv: 500, 1000 or 1500 mg/ kg bw/ day | 10 days | TNFα: LPS↑, LPS+Resv→ | [31] |
| 6-OHDA: 6-hydroxydopamine; ANG II: Angiotensin II; BDL: Bile duct ligation; BLE: bleomycin; COX: Cyclooxygenase; CS: the cigarette smoke; DMN: Dimethylnitrosamine; DSS: dextran sulfate sodium; EAC: Ehrlich ascites carcinoma; EAE: Experimental autoimmune encephalomyelitis; iNOS: inducible Nitrogenoxide Synthase; LPS: Lipopolysacharide; MCT: Monocrotaline; MTX: Methotrexate; NAP: Naphthalene; OBS: Obesity; ODC: Ornithine decarboxylase; OVA: ovalbumin; ST: Steatosis; STZ: Streptozotocin; TNBS: trinitrobenzenesulphonic acid; UVB: Ultraviolet radiation B  ig: intra gastrically; iv: intravenous; ip: intraperitoneally; po: per oral;  Effect are indicated by ↓: reduction; ↑: enhancement; →: no effect. | | | | | |

**References**

1. Bedirli A, Salman B, Pasaoglu H, Ofluoglu E, Sakrak O (2010) Effects of Nuclear Factor-kappaB Inhibitors on Colon Anastomotic Healing in Rats. J Surg Res. DOI:10.1016/j.jss.2010.01.028.

2. Martin AR, Villegas I, Sanchez-Hidalgo M, de la Lastra CA (2006) The effects of resveratrol, a phytoalexin derived from red wines, on chronic inflammation induced in an experimentally induced colitis model. Br J Pharmacol 147: 873-885.

3. Larrosa M, Yanez-Gascon MJ, Selma MV, Gonzalez-Sarrias A, Toti S et al. (2009) Effect of a low dose of dietary resveratrol on colon microbiota, inflammation and tissue damage in a DSS-induced colitis rat model. J Agric Food Chem 57: 2211-2220.

4. Sengottuvelan M, Deeptha K, Nalini N (2009) Influence of dietary resveratrol on early and late molecular markers of 1,2-dimethylhydrazine-induced colon carcinogenesis. Nutrition 25: 1169-1176.

5. Hong SW, Jung KH, Zheng HM, Lee HS, Suh JK et al. (2010) The protective effect of resveratrol on dimethylnitrosamine-induced liver fibrosis in rats. Arch Pharm Research 33: 601-609.

6. Kumar A, Sharma SS (2010) NF-kappaB inhibitory action of resveratrol: A probable mechanism of neuroprotection in experimental diabetic neuropathy. Biochem Biophys Res Commun 394: 360-365.

7. Palsamy P, Subramanian S (2010) Ameliorative potential of resveratrol on proinflammatory cytokines, hyperglycemia mediated oxidative stress, and pancreatic beta-cell dysfunction in streptozotocin-nicotinamide-induced diabetic rats. J Cell Physiol 224: 423-432.

8. Tunali-Akbay T, Sehirli O, Ercan F, Sener G (2010) Resveratrol protects against methotrexate-induced hepatic injury in rats. J Pharm Pharm Sci 13: 303-310.

9. Csiszar A, Labinskyy N, Olson S, Pinto JT, Gupte S et al. (2009) Resveratrol prevents monocrotaline-induced pulmonary hypertension in rats. Hypertension 54: 668-675.

10. Rivera L, Moron R, Zarzuelo A, Galisteo M (2009) Long-term resveratrol administration reduces metabolic disturbances and lowers blood pressure in obese Zucker rats. Biochem Pharmacol 77: 1053-1063.

11. Csiszar A, Labinskyy N, Podlutsky A, Kaminski PM, Wolin MS et al. (2008) Vasoprotective effects of resveratrol and SIRT1: attenuation of cigarette smoke-induced oxidative stress and proinflammatory phenotypic alterations. Am J Physiol Heart Circ Physiol 294: H2721-H2735.

12. Jin F, Wu Q, Lu YF, Gong QH, Shi JS (2008) Neuroprotective effect of resveratrol on 6-OHDA-induced Parkinson's disease in rats. Eur J Pharmacol 600: 78-82.

13. Bujanda L, Hijona E, Larzabal M, Beraza M, Aldazabal P et al. (2008) Resveratrol inhibits nonalcoholic fatty liver disease in rats. BMC Gastroenterol 8: 40.

14. Sener G, Topaloglu N, Ozer SA, Ercan F, Gedik N (2007) Resveratrol alleviates bleomycin-induced lung injury in rats. Pulm Pharmacol Ther 20: 642-649.

15. Ara C, Kirimlioglu H, Karabulut AB, Coban S, Ay S et al. (2005) Protective effect of resveratrol against oxidative stress in cholestasis. J Surg Res 127: 112-117.

16. Cui X, Jin Y, Hofseth AB, Pena E, Habiger J et al. (2010) Resveratrol suppresses colitis and colon cancer associated with colitis. Cancer Prev Res 3: 549-559.

17. El-Mowafy AM, El-Mesery ME, Salem HA, Al-Gayyar MM, Darweish MM (2010) Prominent chemopreventive and chemoenhancing effects for resveratrol: unraveling molecular targets and the role of C-reactive protein. Chemotherapy 56: 60-65.

18. Larrosa M, Tome-Carneiro J, Yanez-Gascon MJ, Alcantara D, Selma MV et al. (2010) Preventive oral treatment with resveratrol pro-prodrugs drastically reduce colon inflammation in rodents. J Med Chem 53: 7365-7376.

19. Sanchez-Fidalgo S, Cardeno A, Villegas I, Talero E, de la Lastra CA (2010) Dietary supplementation of resveratrol attenuates chronic colonic inflammation in mice. Eur J Pharmacol 633: 78-84.

20. Singh UP, Singh NP, Singh B, Hofseth LJ, Price RL et al. (2010) Resveratrol (trans-3,5,4'-trihydroxystilbene) induces silent mating type information regulation-1 and down-regulates nuclear transcription factor-kappaB activation to abrogate dextran sulfate sodium-induced colitis. J Pharmacol Exp Ther 332: 829-839.

21. Yao J, Wang JY, Liu L, Li YX, Xun AY et al. (2010) Anti-oxidant effects of resveratrol on mice with DSS-induced ulcerative colitis. Arch Med Res 41: 288-294.

22. Zhang H, Morgan B, Potter BJ, Ma L, Dellsperger KC et al. (2010) Resveratrol improves left ventricular diastolic relaxation in type 2 diabetes by inhibiting oxidative/nitrative stress. Am J Physiol Heart Circ Physiol 299: H985-H994.

23. Inanaga K, Ichiki T, Matsuura H, Miyazaki R, Hashimoto T et al. (2009) Resveratrol attenuates angiotensin II-induced interleukin-6 expression and perivascular fibrosis. Hypertens Res 32: 466-471.

24. Lee M, Kim S, Kwon OK, Oh SR, Lee HK et al. (2009) Anti-inflammatory and anti-asthmatic effects of resveratrol, a polyphenolic stilbene, in a mouse model of allergic asthma. Int Immunopharmacol 9: 418-424.

25. Sehirli O, Tozan A, Omurtag GZ, Cetinel S, Contuk G et al. (2008) Protective effect of resveratrol against naphthalene-induced oxidative stress in mice. Ecotoxicol Environ Safety 71: 301-308.

26. Li T, Fan GX, Wang W, Li T, Yuan YK (2007) Resveratrol induces apoptosis, influences IL-6 and exerts immunomodulatory effect on mouse lymphocytic leukemia both in vitro and in vivo. Int Immunopharmacol 7: 1221-1231.

27. Norata GD, Marchesi P, Passamonti S, Pirillo A, Violi F et al. (2007) Anti-inflammatory and anti-atherogenic effects of cathechin, caffeic acid and trans-resveratrol in apolipoprotein E deficient mice. Atherosclerosis 191: 265-271.

28. Sharma S, Chopra K, Kulkarni SK (2007) Effect of insulin and its combination with resveratrol or curcumin in attenuation of diabetic neuropathic pain: participation of nitric oxide and TNF-alpha. Phytother Res 21: 278-283.

29. Singh NP, Hegde V, Hofseth LJ, Nagarkatti M, Nagarkatti P (2007) Resveratrol (trans-3,5,4'-trihydroxystilbene) ameliorates experimental allergic encephalomyelitis, primarily via induction of apoptosis in T cells involving activation of aryl hydrocarbon receptor and estrogen receptor. Mol Pharmacol 72: 1508-1521.

30. Afaq F, Adhami VM, Ahmad N (2003) Prevention of short-term ultraviolet B radiation-mediated damages by resveratrol in SKH-1 hairless mice. Toxicol Appl Pharmacol 186: 28-37.

31. Liu HS, Pan CE, Yang W, Liu XM (2003) Antitumor and immunomodulatory activity of resveratrol on experimentally implanted tumor of H22 in Balb/c mice. World J Gastroenterol 9: 1474-1476.
